# The Demographics of Canine Hip Dysplasia in the United States and Canada

**DOI:** 10.1155/2017/5723476

**Published:** 2017-03-12

**Authors:** Randall T. Loder, Rory J. Todhunter

**Affiliations:** ^1^Department of Orthopaedic Surgery, Indiana University School of Medicine and James Whitcomb Riley Children's Hospital, Indianapolis, IN 46202, USA; ^2^Department of Clinical Sciences, College of Veterinary Medicine, Cornell University, Ithaca, NY 14853-6401, USA

## Abstract

Canine hip dysplasia (CHD) is a common problem in veterinary medicine. We report the demographics of CHD using the entire hip dysplasia registry from the Orthopedic Foundation for Animals, analyzing differences by breed, sex, laterality, seasonal variation in birth, and latitude. There were 921,046 unique records. Each dog was classified using the American Kennel Club (AKC) and Fédération Cynologique Internationale (FCI) systems. Statistical analysis was performed with bivariate and logistic regression procedures. The overall CHD prevalence was 15.56%. The OR for CHD was higher in females (1.05), those born in spring (1.14) and winter (1.13), and those in more southern latitudes (OR 2.12). Within AKC groups, working dogs had the highest risk of CHD (OR 1.882) with hounds being the reference group. Within FCI groups, the pinscher/molossoid group had the highest risk of CHD (OR 4.168) with sighthounds being the reference group. The similarities between CHD and DDH are striking. Within DDH there are two different types, the typical infantile DDH and the late onset adolescent/adult acetabular dysplasia, with different demographics; the demographics of CHD are more similar to the later onset DDH group. Comparative studies of both disorders should lead to a better understanding of both CHD and DDH.

## 1. Introduction

Canine hip dysplasia (CHD) is a well-known disorder in veterinary medicine [1–4], especially amongst certain breeds. The human counterpart of CHD, developmental dysplasia of the hip (DDH), is also a well-known problem with differences in prevalence by race/ethnicity [5], analogous to breed differences in CHD. Comprehensive literature reviews of DDH have shown various demographic patterns regarding sex, laterality, latitude, and seasonal variation in birth month [5, 6]. Variation in birth month/season has been described in a few small series of CHD [7–12]. There has been no study of the demographics of CHD using a large data set. The purpose of this study was to investigate the demographics of CHD using a large North American data base and analyze the differences by breed, sex, laterality, seasonal variation in birth, and latitude. Comparison with the demographics of DDH may shed further light on the etiology of both conditions and specifically support the use of CHD as an animal model for DDH, as well as DDH pointing towards further comparative research areas in CHD.

## 2. Materials and Methods

### 2.1. Data Source

The data for this study was the complete hip dysplasia registry (both public and private) collected by the Orthopedic Foundation for Animals (OFA) through April 2015. There were a total of 1,430,979 records. The OFA hip score uses the American Veterinary Medical Association grading system: 1 = excellent, 2 = good, 3 = fair, 4 = borderline CHD, 5 = mild CHD, 6 = moderate CHD, and 7 = severe CHD. These scores were divided into two groups: those with CHD (scores 5–7) and those without CHD (scores 1–3); the borderline score of 4 was excluded. Duplicate records, feline cases, and those with an indeterminate score were deleted. The country of origin was known in 1,130,478 dogs; the vast majority (1,121,961–99.25%) were from the USA (1,046,249) or Canada (75,712). Dogs less than 24 or greater than 60 months of age at the time of the radiograph were next deleted, leaving 921,046 unique records which are the data for this study.

### 2.2. Data Groups

Each dog was classified into related breed groups using both the American Kennel Club (AKC) (http://www.akc.org) [13] and Fédération Cynologique Internationale (FCI) (http://www.fci.be/en/Nomenclature) [14] systems. Each dog was separately given an AKC and FCI group designation and analyzed separately; the two different systems were not merged. Dogs in each of these groups are relatively similar genetically [15, 16] and thus could be expected to respond to environmental triggers similarly, compared to dogs that do not share a common genetic background. The AKC categories are herding, hound, working, sporting, nonsporting, terrier, toy, native, hybrid, and miscellaneous groups. The FCI categories are (1) sheep and cattle dogs; (2) pinscher, schnauzer, molossoid, and Swiss mountain and Swiss cattle dogs; (3) terriers; (4) dachshunds; (5) spitz and primitive dogs; (6) scent hounds; (7) pointers; (8) retrievers, flushers, and water dogs; (9) companion and toy dogs; and (10) sighthounds.

The variables analyzed were sex, breed, season of birth, hip score, and latitude. Season of birth was arbitrarily defined as follows: winter, December through February, spring, March through May, summer, June through August, and autumn, September through November. Each state and province was grouped by latitude. The latitude where each dog was living at the time of the radiograph was placed into 4 groups defined as (1) <30°N, (2) 30–39°N, (3) 40–49°N, and (4) >50°N. Those <30°N were Florida, Hawaii, Louisiana, Puerto Rico, Virgin Islands, and Guam. Those 30–39°N were Alabama, Arkansas, Arizona, California, Colorado, District of Columbia, Delaware, Georgia, Indiana, Kansas, Kentucky, Maryland, Missouri, Mississippi, North Carolina, New Mexico, Nevada, Oklahoma, South Carolina, Tennessee, Texas, Virginia, and West Virginia. Those 40–49°N were the states of Connecticut, Iowa, Idaho, Illinois, Maryland, Maine, Michigan, Minnesota, Montana, Nebraska, New Hampshire, New Jersey, New York, Ohio, Oregon, Pennsylvania, Rhode Island, South Dakota, Utah, Vermont, Washington, Wisconsin, and Wyoming and the provinces of New Brunswick, Newfoundland, Nova Scotia, Ontario, Prince Edward Island, and Quebec. Those >50°N were the state of Alaska and the provinces of Alberta, British Columbia, Manitoba, Northwest Territories, Saskatchewan, and Yukon Territory. Although a few of the states and provinces straddle these latitude lines, each state/province was placed into the group corresponding to the major population areas.

### 2.3. Statistical Analysis

Demographic variables were first analyzed using bivariate analyses (Pearson's *χ*^2^ test) to determine differences between those with and without CHD. Next, binary multivariate logistic regression analyses were performed to determine adjusted odds ratios (OR) and 95% [upper, lower] confidence intervals of a dog having CHD. While the American Veterinary Medical Association grading system is a numerical value, it is not a continuous variable such as the Norberg angle, but rather a categorical ordinal variable determined by subjective criteria (http://www.ofa.org/hd_grades.html – hip dysplasia, OFA X-ray procedures). For this reason, CHD grade was considered to be a categorical variable. All statistical analyses were performed with Systat™ 10 software (Chicago, IL, 2000), and *p* < 0.05 was considered statistically significant.

## 3. Results

### 3.1. Overall Results

**Figure 1:**
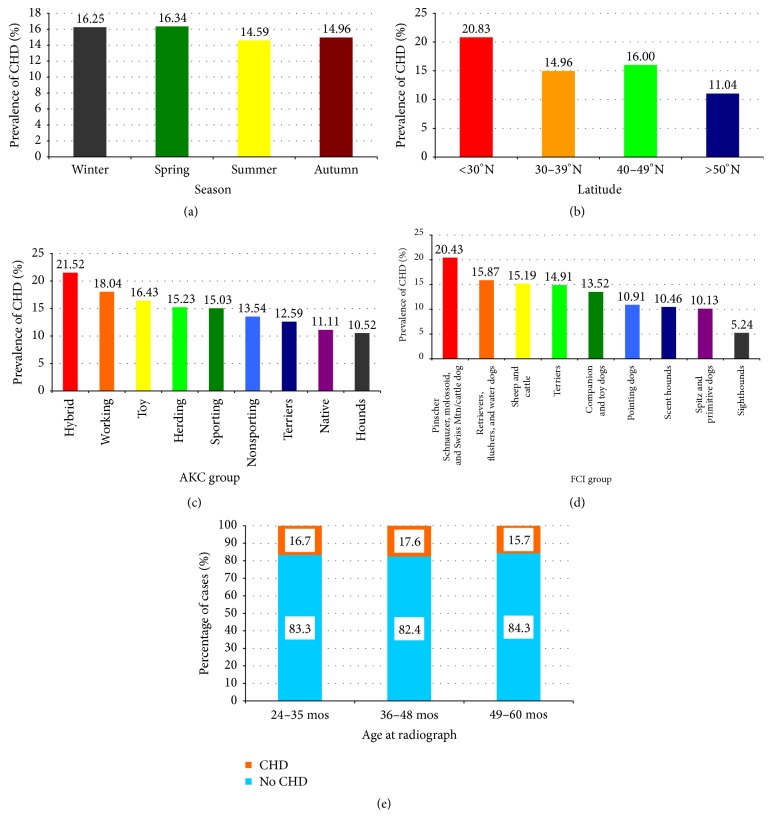
Prevalence of CHD by various demographic parameters. (a) By season of birth. (b) By latitude. (c) By AKC groups. (d) By FCI groups. (e) By age at time of radiograph. The numbers in the boxes are the percentage within each column bar.

The hip dysplasia scores were 1 in 74,931 dogs; 2 in 601,893; 3 in 95,154; 4 in 6,772; 5 in 86,321; 6 in 47,971; and 7 in 8,004, resulting in an overall CHD prevalence of 15.56%. There was significant variability in the prevalence of CHD by AKC and FCI groups, gender, latitude, and season of birth (Table 1). CHD was overall slightly more common in females, those born in spring and winter (Figure 1(a)), and those born in the more southern latitudes (Figure 1(b)). Within AKC groups, CHD was most prevalent in hybrid breeds (21.5%) and least prevalent in hounds (10.5%) (Figure 1(c)). Within FCI groups, it was most prevalent in group 2 (pinscher, schnauzer, molossoid, and Swiss mountain/Swiss cattle dogs) (20.4%) and least common in group 10 (sighthounds) (5.2%) (Figure 1(d)). Although there was a statistically significant difference in the prevalence of CHD by age at the time of radiography (Figure 1(e)), the variability was less than 2% and considered to not be clinically significant, especially since the oldest group of dogs had a lower prevalence of CHD than the youngest cohort. Age was thus deleted from all further analyses. There was significant variation by individual breeds. The prevalence of CHD by breeds in this study is very similar to that given on the OFA website http://www.ofa.org, even though dogs outside of Canada or the USA were excluded in our study. The complete CHD prevalence data set is given in Supplemental Table 1 in Supplementary Material available online at https://doi.org/10.1155/2017/5723476; the highest prevalence was in the bulldog (77.7%) and the lowest in the Italian greyhound (0.0%).

### 3.2. Results by Demographic Parameters

The overall OR for CHD was higher in females (1.05 [1.064, 1.039]; *p* < 10^−6^), those born in spring (1.143 [1.16, 1.13]; *p* < 0.004), and those living in more southern latitudes (<30°N) (OR 2.12; [2.21, 2.04]; *p* < 10^−6^). These results from the composite data set obviously reflect the proportion of breeds in the OFA database and could likely be different if the breed composition differed. Therefore, analyses for each AKC and FCI group, as well as individual breeds, were performed (Table 2). Due to small numbers in certain groups, those in the native, hybrid, and miscellaneous were excluded when analyzing by AKC groups and the dachshunds when analyzing by FCI groups. Within AKC groups, working dogs had the highest risk of CHD (OR 1.882) with hounds being the reference group. Within FCI groups, group 2 (pinscher, schnauzer, molossoid, and Swiss mountain/Swiss cattle dogs) had the highest risk of CHD (OR 4.168) with sighthounds being the reference group. Those born in spring had the highest risk of CHD (OR 1.14) as well as those living in latitudes < 30°N (OR 2.1), with a minimally higher risk in females (OR 1.05).

### 3.3. Results by AKC and FCI Groups

Analyses by each of the AKC and FCI groups were next performed (Table 3). Again, many of the groups showed an increase in CHD in those living in latitudes <30°N, except for toy dogs (where the opposite was noted with a higher risk in the most northern latitudes >50°N); hounds had no variation in CHD by latitude. When there was an increased OR by season of birth, winter and spring seasons most commonly demonstrated the increased risk with a few demonstrating an autumn increase; no group demonstrated a summer increase. A few groups demonstrated an increased CHD risk in females (AKC herding, working and sporting groups and FCI sheep/cattle and pinscher groups); sighthounds had an increased risk in male dogs.

Analyses within subgroups of AKC and FCI groups (Supplemental Table 2) as well as the most common 25 breeds in the data set (Supplemental Table 3) were also performed. Here again, similar findings are as seen for individual AKC and FCI groups. The detailed ORs of CHD for all dogs with *n* > 1000 as well as all dogs with *n* > 100 and a CHD prevalence of >15% (the median value) are given in Supplemental Table 4.

### 3.4. Severity and Laterality of CHD

**Figure 2:**
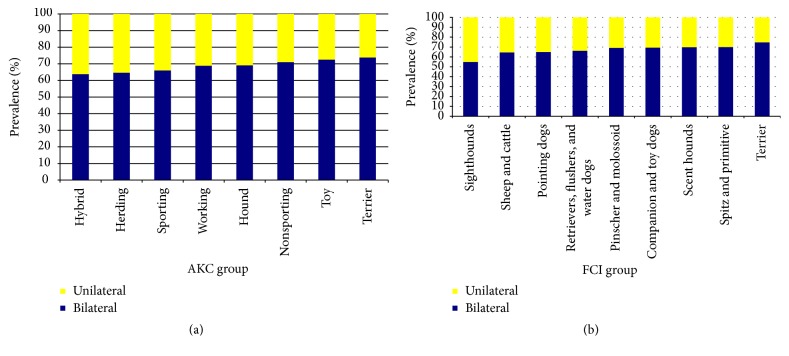
Unilateral and bilateral involvement in CHD. (a) By AKC group. (b) By FCI group.

For those dogs with CHD, severity of the CHD was analyzed (Table 4). Severe CHD (score of 7) was more common in those with bilateral involvement, AKC groups of herding and working dogs, FCI groups of pinscher and sheep/cattle dogs, those living in the most southern latitudes (<30°N), and those born in spring. Males had a slightly higher proportion of severe CHD. Regarding unilateral or bilateral involvement, bilateral disease was most prevalent in terriers and least prevalent in hybrid dogs within AKC groups (Figure 2(a)); bilateral disease was most prevalent in terriers and least prevalent in sighthounds within FCI groups (Figure 2(b)).

## 4. Discussion

Limitations of this study need to be acknowledged. Although we used a very large data set, it may not give the true prevalence of CHD, since it only represents the data on those dogs whose radiographs were submitted to the OFA. This predisposes to selection bias as it is not a truly random sample of the canine population [17]. Determination of the “true” prevalence would require a prospective radiographic exam between 2 and 5 years of age of every dog consecutively born, with a population of at least 1 million. Obviously such a study is impossible to perform. The OFA data set is therefore likely the best that can be presently obtained in the North America with the possible exception of the PennHIP™.

With these limitations in mind, there are several important findings. CHD is slightly more common in females, but with a large variation, ranging from 3.36 times more frequent in female Polish Tatra Sheepdogs to 1.63 times more frequent in male Afghan Hounds (Supplemental Table 1). CHD prevalence varies by breed, which was again demonstrated in this study, ranging from 77.7% in the bulldog to 0% in the Italian Greyhound. Many breeds demonstrated a mild increase in risk for CHD when born in winter and spring. CHD was unilateral in 33% of all dogs with CHC. Unilateral involvement was more common in herding/sporting dogs and they had lower hip dysplasia scores. Finally, a new finding is that the prevalence of CHD is more common in dogs living in more southern latitudes.

This study confirms the marked variability in CHD prevalence by breed. In France, the highest prevalence of CHD was in the Cane Corso (59.7%) and the lowest in the Siberian Husky (3.9%) [18]. In a national Veterinary Medical Database from the entire USA [19], the OR of CHD was 10.2 in the Kuvasz with mixed breed dogs being the reference group. In a more recent study using the Veterinary Medical Database [20] the highest prevalence of CHD was 17.16% in the Newfoundland and 0.12% in the Scottish Terrier. In USA veterinary teaching hospitals, the prevalence of CHD was highest in the Rottweiler (35.4%) and lowest in the miniature schnauzer dogs (1.5%) [21]. In a Norwegian study comprised of Newfoundland, Leonberger, Labrador Retriever, and Irish Wolfhounds (*n* = 501), the highest prevalence of CHD was in the Newfoundland and the lowest in the Irish Wolfhound (OR 0.22 that of the Newfoundland) [22]. In Turkey, a study of 484 dogs from 7 different breeds revealed the highest prevalence in Doberman Pinschers (70.6%) and the lowest in Golden Retrievers (50%); the prevalence in Doberman Pinschers in this study in North America was low at 5.1%. It must be remembered that many of these studies used a different grading system than the OFA scores; however, it still confirms marked variability within breeds within each study.

The quoted prevalence of CHD is frequently different between different studies for a particular breed. When comparing the data of Witsberger et al. [20] to ours, the prevalence of CHD for the Newfoundland was 17.2% versus 20.0%, Saint Bernard 14.7% versus 36.8%, Rottweiler 10.3% versus 12.5%, German Shepherd 10.3% versus 16.3%, Golden Retriever 8.5% versus 14.9%, Labrador Retriever 7.4% versus 9.2%, Bulldog 4.4% versus 68.9%, Doberman Pinscher 1.3% versus 5.1%, and Greyhound 0.4% versus 2.1%, respectively. This demonstrates that the sampling technique/composition of the data set markedly impacts the prevalence value as previously mentioned. Prevalence amongst each breed within a country, or region, is likely a result of gene flow, bottle necks, popular sire effects, and the efforts of individuals and breed clubs to impact the prevalence and severity of CHD in a particular breed.

We noted a slight increase in CHD in females with marked differences by breed. Several studies noted no sex difference in the prevalence of CHD. In Norway, Turkey, and the United Kingdom no sex differences were noted for the various breeds studied [22–25]. In Sweden, CHD was 1.14 times more common in female German Shepherds compared to males [26]. In the United States, sex differences were noted in Golden Retrievers [27]; the prevalence of CHD was 5.1% in intact males, 10.3% in males neutered early, 0% in males neutered late, 39% in intact females, 4.5% in females neutered early, and 0% in females neutered late. The status of neutering in the OFA registry is not given, so we cannot compare our findings to those of Torres de la Riva [27].

The prevalence of unilateral CHD was 33% in this study. The prevalence of unilateral CHD was 35% in a New York study of 1022 dogs consisting of Labrador Retrievers, Golden Retrievers, German Shepherds, and crossbreeds [2]. In Pennsylvania, it was 6% in 133 Greyhounds. A recent study of multiple breeds from Italy noted an overall percentage of unilateral CHD of 31.5% [28], strikingly similar to the 33% in this study and the 35% of Lust et al. [2]. This is the first study to investigate the proportion of unilateral CHD by AKC/FCI groups; for AKC groups it was highest in herding dogs (35.4%) and lowest in terriers (27.5%); for FCI groups it was highest in sheep/cattle dogs (35.4%) and lowest in terriers (25.1%) (Table 1).

Few studies discuss season of birth and CHD. In Norway [29], the OR for CHD (Newfoundland, Leonberger, Labrador Retriever, and Irish Wolfhounds) was 3.94 times higher in autumn and 1.85 times higher in winter compared to spring. In another Norwegian study [9], pointers had an increase in CHD in those born in August to February, Labrador Retrievers September to February, with no seasonal effect on CHD in German Shepherds or Golden Retrievers. In Finland [7], German Shepherds born in spring or summer had less CHD. In England [10], Labrador Retrievers and Gordon Setters had less CHD when born in July through October. In New Zealand [8], Labrador Retrievers and Rottweilers had less CHD when born in autumn, but no seasonal variation was observed for German Shepherds or Golden Retrievers. In aggregate, the previous studies in the Northern Hemisphere noted that dogs born in autumn and/or winter months demonstrate a higher prevalence of CHD. In this study we noted an increase of CHD primarily in winter and spring months. When reviewing the data from Supplemental Table 3, 563,403 of the 619,825 dogs (81.4%) showed a seasonal variation. Of these 536,403, 313,202 (55.6%) had the highest percentage in winter, 229,925 (40.8%) in spring, and 20,276 (3.6%) in autumn.

There are several postulated reasons for seasonal differences in CHD. One is the relationship between hip muscle development and season. The most critical time for canine hip joint development is between 3 and 9 months of age [8, 30]; cage confinement during this crucial period has a protective effect on the hip [30]. The proposed explanation is that puppies born in winter spend more time in cages/indoors than in free activities, and indoor confinement may keep the hips in flexion and abduction lessening the development of CHD [29]. The same has been noted in human DDH, where carrying the infant in positions of hip abduction and flexion reduces the incidence of DDH [31–35] while swaddling in extension increases the incidence of DDH [5, 36, 37]. Our results refute a winter protective effect in CHD. A second explanation is that puppies born in late autumn or early winter, compared to those born in spring or early summer, do not get as much physical exercise. Puppies getting less physical exercise may develop weaker hip musculature than those with a lot of outdoor activity, which when combined with rapid skeletal growth results in weakened constraints on the hip, subsequent subluxation, and CHD [8, 22, 29, 30]. This can explain the increase in CHD in dogs born in late autumn/early winter and corroborates the findings from New Zealand, England, and our study, while conflicting with the data from Norway, Finland, and Sweden.

Another postulated mechanism for CHD seasonal variation is diet and weight gain in puppies. Dogs with limited weight gain in early life have a lower prevalence of CHD [2, 22, 29, 38, 39]. In cold winter months dogs have increased food intake [40, 41], and if not accompanied by an increase in energy consumption (e.g., activity), the dog will gain weight. Increased body weight increases the stress across the developing hip joint leading to subluxation [17, 42, 43]. Vitamin D plays a role in DDH, as humans with homozygosity for the mutant Taq1 vitamin D receptor *t* allele demonstrate increased acetabular dysplasia [44]. Vitamin D levels may vary by season due to seasonal variation in vitamin D dietary content in both humans and animals [45–52]. Low vitamin D levels and increased body fat in winter may result in more CHD. Finally, various dietary factors differ by season and could result in seasonal differences in hormones in milk (vitamin D, relaxin, and vitamin C) and secondarily influence hip development [52–57].

This is the first description of an increased prevalence of CHD in more southern latitudes. This was true even when multivariate regression logistic analysis was performed adjusting for breed group, gender, and season of birth. One potential explanation is that the generally warmer climate in more southern latitudes may result in a general increase in physical activity at all times, with the hips being less abducted and flexed, resulting in more CHD. Another potential explanation is that the gene pools may be different in different latitudes. Finally, other environmental factors such as diet as discussed above may be involved, resulting in increased CHD. Perhaps the dogs in the more southern latitudes are heavier and place more stress across the hip. It could also be that the dogs in the warmer more southern latitudes grow more rapidly early in life, which is a well-known contributing factor to CHD [38, 39]. This finding and potential explanations will require further study.

There are marked differences and similarities between DDH and CHD (Table 5). The most striking is the difference in incidence/prevalence by race/breed. Prevalence/incidence variation in humans is higher (950-fold difference in Native Americans compared to Africans in Africa) than canines (96-fold difference in the bulldog compared to the whippet) (Supplemental Table 1). DDH occurs predominantly in females (75%) for all races [5], while for CHD the prevalence was only slightly higher in females compared to males (Table 1). However there are large sex variations in CHD which ranged from 3.4 times more frequent in female Polish Tatra Sheepdogs to 1.6 times more frequent in male Afghan Hounds. DDH is usually unilateral (63.4%) [5] compared to CHD which is usually bilateral (67%). DDH demonstrates a seasonal variation in ~91.0% of cases [6], and 81.4% in CHD, which is remarkably similar. DDH was most prevalent when the baby was born in winter months (70.3%); CHD was most prevalent when the puppy was born in winter and spring. DDH is more common in northern latitudes, while CHD is more common in southern latitudes [5, 6]. This latitudinal difference has also been noted in children with Perthes' disease [58]. Within DDH there are two different types, the typical infantile DDH and the late onset adolescent/adult acetabular dysplasia [59]. The older group, when compared to the infantile group, demonstrated a lower female predominance (88.0 versus 98.0%) with more bilateral involvement (61.2% versus 45.1%). Our findings in CHD more closely mirror the demographics of DDH in the late onset group.

In conclusion, the prevalence of CHD differed markedly by breed, having a slight female predominance but with significant variability by breed, was unilateral in about one-third of cases, and often demonstrated a seasonal variation with a mild increase when the dog was born in spring and winter months. Most interestingly, CHD was more prevalent in the more southern latitudes. This information is important to owners/breeders, suggesting that monitoring of puppies for signs of CHD should be undertaken during the birth months when there is an increased OR of CHD for those affected breeds and/or AKC groups, especially in more southern latitudes. The similarities between CHD and DDH are striking, especially late onset DDH, and suggest that comparative studies of both disorders should lead to a better understanding of a problem that leads to debilitating hip osteoarthritis in both canines and humans.

## Supplementary Material

The supplementary material in these gives detailed results of many of the analyses done, such as detailed prevalence or odds ratios for many specific breeds and groups. This is for the reader interested in more information.

## Figures and Tables

**Table 1:** Prevalence of CHD by demographic variables.

Parameter	All	CHD versus no CHD					Bilateral versus unilateral CHD					Right versus left unilateral CHD				
		Dogs without CHD	Dogs with CHD	% CHD	% without CHD	*p* value	Bilateral CHD	Unilateral CHD	% bilateral	% unilateral	*p* value	Left CHD	Right CHD	% left	% right	*p* value
																
*All dogs*	914,274	771,978	142,296	15.56	84.44	—	95,376	46,918	67.03	32.97	—	21,657	18,140	54.42	45.58	—
*Sex*																
Female	582,990	490,884	92,106	15.80	84.20	<10^−6^	17,079	50,189	65.97	34.03	<10^−6^	8,039	6,444	55.51	44.49	0.001
Male	331,281	281,091	50,190	15.15	84.85		29,839	92,105	67.60	32.40		13,618	11,696	53.80	46.20	
*AKC group*																
Herding	181,497	153,857	27,640	15.23	84.77	<10^−6^	9,770	27,639	64.65	35.35	<10^−6^	4,346	3,922	52.56	47.44	<10^−6^
Hound	24,017	21,490	2,527	10.52	89.48		782	2,527	69.05	30.95		359	307	53.90	46.10	
Working	217,397	178,178	39,219	18.04	81.96		12,215	39,219	68.85	31.15		5,076	4,898	50.89	49.11	
Sporting	404,008	343,284	60,724	15.03	84.97		20,628	60,723	66.03	33.97		10,006	7,694	56.53	43.47	
Nonsporting	51,153	44,226	6,927	13.54	86.46		2,005	6,927	71.06	28.94		1,031	238	81.25	18.75	
Terrier	19,234	16,812	2,422	12.59	87.41		633	2,422	73.86	26.14		341	238	58.89	41.11	
Toy	11,005	9,197	1,808	16.43	83.57		497	1,808	72.51	27.49		277	194	58.81	41.19	
Native	36	32	4	11.11	88.89		2	4	50.00	50.00		1	1	50.00	50.00	
Hybrid	2,514	1,973	541	21.52	78.48		196	541	63.77	36.23		117	66	63.93	36.07	
Miscellaneous	3,413	2,929	484	14.18	85.82		190	484	60.74	39.26		103	77	57.22	42.78	
*FCI group*																
Sheep and cattle	185,969	157,713	28,256	15.19	84.81	<10^−6^	18,250	10,005	64.59	35.41	<10^−6^	4,465	4,004	52.72	47.28	<10^−6^
Pinscher Schnauzer, molossoid, and Swiss Mtn/cattle dog	176,144	140,164	35,980	20.43	79.57		24,919	11,061	69.26	30.74		4,571	4,462	50.60	49.40	
Terrier	14,542	12,374	2,168	14.91	85.09		1,623	545	74.86	25.14		291	209	58.20	41.80	
Dachshund	70	63	7	10.00	90.00		7	0	100.00	0.00		0	0	—	—	
Spitz and primitive	64,683	58,132	6,551	10.13	89.87		4,588	1,963	70.04	29.96		915	740	55.29	44.71	
Scent hounds	16,509	14,782	1,727	10.46	89.54		1,207	520	69.89	30.11		238	200	54.34	45.66	
Pointing dogs	71,170	63,403	7,767	10.91	89.09		5,045	2,721	64.96	35.04		1,293	1,073	54.65	45.35	
Retrievers, flushers, and water dogs	340,551	286,489	54,062	15.87	84.13		35,797	18,265	66.21	33.79		8,912	6,746	56.92	43.08	
Companion and toy dogs	37,085	32,071	5,014	13.52	86.48		3,484	1,530	69.49	30.51		815	586	58.17	41.83	
Sighthounds	5,129	4,860	269	5.24	94.76		148	121	55.02	44.98		48	54	47.06	52.94	
*Latitude*																
<30°N	43,929	34,779	9,150	20.83	79.17	<10^−6^	6,428	2,721	70.26	29.74	<10^−6^	1,172	1,074	52.18	47.82	0.0037
30–39°N	404,336	343,861	60,475	14.96	85.04		40,594	19,880	67.13	32.87		9,131	7,865	53.72	46.28	
40–49°N	425,790	357,670	68,120	16.00	84.00		45,283	22,837	66.48	33.52		10,650	8,619	55.27	44.73	
≥50°N	37,506	33,364	4,142	11.04	88.96		2,777	1,365	67.04	32.96		655	546	54.54	45.46	
*Season of birth*																
Autumn	215,003	182,842	32,161	14.96	85.04	<10^−6^	21,362	10,798	66.42	33.58	0.0016	5,020	4,099	55.05	44.95	0.10
Winter	218,849	183,276	35,573	16.25	83.75		23,695	11,878	66.61	33.39		5,582	4,555	55.07	44.93	
Spring	255,064	213,377	41,687	16.34	83.66		28,146	13,540	67.52	32.48		6,151	5,278	53.82	46.18	
Summer	225,358	192,483	32,875	14.59	85.41		22,173	10,702	67.45	32.55		4,904	4,208	53.82	46.18	

**(a):** 

By AKC group			
	OR	95% CI	*p* value
*Sex*			
Female	1.056	(1.069, 1.044)	<10^−6^
Male	1.0 R	—	—
*Season of birth*			
Autumn	1.025	(1.042, 1.008)	<10^−6^
Winter	1.131	(1.149, 1.112)	0.081
Spring	1.146	(1.165, 1.128)	<10^−6^
Summer	1.0 R	—	—
*Latitude*			
<30°N	2.116	(2.203, 2.034)	<10^−6^
30–39°N	1.428	(1.477, 1.381)	<10^−6^
40–49°N	1.552	(1.605, 1.501)	<10^−6^
≥50°N	1.0 R	—	—
*AKC group*			
Herding	1.535	(1.602, 1.470)	<10^−6^
Toy	1.675	(1.788, 1.570)	<10^−6^
Working	1.882	(1.965, 1.804)	<10^−6^
Sporting	1.504	(1.569, 1.442)	<10^−6^
Nonsporting	1.348	(1.415, 1.284)	<10^−6^
Terrier	1.236	(1.311, 1.164)	<10^−6^
Hound	1.0 R	—	—

**(b):** 

By FCI group			
	OR	95% CI	*p* value
*Sex*			
Female	1.053	(1.065, 1.040)	<10^−6^
Male	1.0 R	—	—
*Season of birth*			
Autumn	1.021	(1.038, 1.004)	0.016
Winter	1.124	(1.142, 1.105)	<10^−6^
Spring	1.143	(1.161, 1.125)	<10^−6^
Summer	1.0 R	—	—
*Latitude*			
<30°N	2.047	(2.13, 1.967)	<10^−6^
30–39°N	1.410	(1.458, 1.363)	<10^−6^
40–49°N	1.546	(1.599, 1.496)	<10^−6^
≥50°N	1.0 R	—	—
*FCI group*			
Sheep and cattle	3.229	(3.653, 2.854)	<10^−6^
Pinscher schnauzer, molossoid, and Swiss Mtn/cattle dog	4.618	(5.224, 4.082)	<10^−6^
Terrier	3.163	(3.605, 2.774)	<10^−6^
Spitz and primitive	2.059	(2.334, 1.816)	<10^−6^
Scent hounds	2.096	(2.393, 1.836)	<10^−6^
Pointing dogs	2.184	(2.473, 1.927)	<10^−6^
Retrievers, flushers, and water dogs	3.386	(3.830, 2.994)	<10^−6^
Companion and toy dogs	2.824	(3.204, 2.489)	<10^−6^
Sighthounds	1.0 R	—	—

**Table 3:** Odds ratios of CHD for each AKC/FCI group by sex, season of birth, and latitude^*∗*^.

		Sex		Latitude						Season of birth					
	*n*	Female	*p* value	(<30°N)	*p* value	(30–39°N)	*p* value	(40–49°N)	*p* value	Autumn	*p* value	Winter	*p* value	Spring	*p* value
		OR (95% CI)		OR (95% CI)		OR (95% CI)		OR (95% CI)		OR (95% CI)		OR (95% CI)		OR (95% CI)	
*By AKC group*															
Herding	180,911	1.14 (1.171, 1.110)	**<10** ^−**6**^	2.129 (2.323, 1.052)	**<10** ^−**6**^	1.383 (1.489, 1.285)	**<10** ^−**6**^	1.531 (1.648, 1.422)	**<10** ^−**6**^	0.996 (1.035, 0.958)	0.83	1.148 (1.191, 1.107)	**<10** ^−**6**^	1.102 (1.141, 1.063)	**<10** ^−**6**^
Toy	11,011	0.948 (1.052, 0.853)	0.31	0.457 (0.620, 0.339)	**<10** ^−**6**^	0.54 (0.678, 0.430)	**<10** ^−**6**^	0.499 (0.628, 0.397)	**<10** ^−**6**^	0.918 (1.062, 0.794)	0.25	1.073 (1.239, 0.930)	0.34	1.041 (1.200, 0.903)	0.58
Working	216,599	1.069 (1.903, 1.045)	**<10** ^−**6**^	2.345 (2.519, 2.182)	**<10** ^−**6**^	1.489 (1.584, 1.401)	**<10** ^−**6**^	1.558 (1.657, 1.465)	**<10** ^−**6**^	0.979 (1.011, 0.948)	0.19	1.075 (1.109, 1.041)	**0.000008**	1.146 (1.182, 1.111)	**<10** ^−**6**^
Sporting	402,911	1.027 (1.046, 1.009)	**0.0038**	2.179 (2.328, 2.038)	**<10** ^−**6**^	1.523 (1.609, 1.441)	**<10** ^−**6**^	1.7 (1.796, 1.609)	**<10** ^−**6**^	1.082 (1.111, 1.056)	**<10** ^−**6**^	1.189 (1.219, 1.159)	**<10** ^−**6**^	1.195 (1.224, 1.167)	**<10** ^−**6**^
Nonsporting	51,045	0.988 (1.042, 0.938)	0.94	1.588 (1.881, 1.341)	**<10** ^−**6**^	1.258 (1.443, 1.096)	**0.0011**	1.266 (1.454, 1.103)	**0.0008**	0.975 (1.046, 0.938)	0.19	1.004 (1.081, 0.932)	**0.000008**	1.067 (1.146, 0.994)	**<10** ^−**6**^
Terriers	19,156	0.978 (1.071, 0.894)	0.63	2.489 (3.281, 1.887)	**<10** ^−**6**^	1.414 (1.776, 1.126)	**0.0028**	1.492 (1.870, 1.190)	**0.0005**	0.987 (1.118, 0.872)	0.84	1.102 (1.245, 0.975)	0.12	1.082 (1.216, 0.963)	0.19
Hounds	23,974	1.024 (1.114, 0.941)	0.58	1.31 (1.748, 0.980)	0.067	1.044 (1.336, 0.815)	0.74	1.165 (1.493, 0.909)	0.23	0.99 (1.115, 0.879)	0.87	0.933 (1.046, 0.832)	0.23	0.92 (1.031, 0.820)	0.15
*By FCI group*															
Sheep and cattle	185,366	1.138 (1.169, 1.109)	**<10** ^−**6**^	2.105 (1.295, 1.931)	**<10** ^−**6**^	1.371 (1.475, 1.274)	**<10** ^−**6**^	1.512 (1.623, 1.405)	**<10** ^−**6**^	0.987 (1.026, 0.951)	0.51	1.145 (1.187, 1.105)	**<10** ^−**6**^	1.098 (1.137, 1.060)	**<10** ^−**6**^
Pinscher schnauzer, molossoid, and Swiss Mtn/cattle dog	175,562	1.078 (1.104, 1.053)	**<10** ^−**6**^	2.227 (2.409, 2.059)	**<10** ^−**6**^	1.52 (1.628, 1.419)	**<10** ^−**6**^	1.626 (1.743, 1.518)	**<10** ^−**6**^	1.006 (1.041, 0.972)	0.74	1.076 (1.113, 1.041)	**0.00002**	1.168 (1.207, 1.131)	**<10** ^−**6**^
Terrier	14,507	1.013 (1.115, 0.921)	0.79	2.131 (2.864, 1.585)	**0.000001**	1.287 (1.651, 1.003)	**0.047**	1.412 (1.810, 1.102)	**0.0064**	0.999 (1.142, 0.875)	0.99	1.125 (1.281, 0.988)	0.076	1.089 (1.233, 0.961)	0.18
Spitz and primitive	64,393	1.014 (1.069, 0.961)	0.62	1.709 (2.022, 1.444)	**<10** ^−**6**^	1.121 (1.263, 0.995)	0.06	1.164 (1.311, 1.033)	**0.012**	1.007 (1.081, 0.938)	0.85	1.008 (1.084, 0.937)	0.84	0.92 (0.992, 0.853)	0.031
Scent hounds	16,490	1.095 (1.215, 0.987)	0.085	1.15 (1.573, 0.841)	0.38	0.911 (1.192, 0.700)	0.50	0.922 (1.212, 0.702)	0.56	0.97 (1.121, 0.839)	0.68	1.09 (1.254, 0.947)	0.23	0.939 (1.074, 0.820)	0.36
Pointing dogs	70,962	0.998 (1.048, 0.951)	0.93	1.412 (1.722, 1.164)	**0.0005**	1.179 (1.384, 1.004)	**0.045**	1.288 (1.509, 1.098)	**0.0018**	1.124 (1.207, 1.046)	**0.0014**	1.257 (1.346, 1.173)	**<10** ^−**6**^	1.184 (1.260, 1.112)	**<10** ^−**6**^
Retrievers, flushers, and water dogs	339,651	1.015 (1.035, 0.995)	0.14	2.305 (2.471, 2.149)	**<10** ^−**6**^	1.578 (1.672, 1.488)	**<10** ^−**6**^	1.781 (1.886, 1.681)	**<10** ^−**6**^	1.044 (1.073, 1.016)	**0.0021**	1.15 (1.182, 1.120)	**<10** ^−**6**^	1.188 (1.213, 1.158)	**<10** ^−**6**^
Companion and toy dogs	37,025	0.99 (1.054, 0.930)	0.75	1.02 (1.234, 0.850)	0.83	0.958 (1.102, 0.832)	0.55	0.97 (1.117, 0.842)	0.67	0.938 (1.054, 0.930)	0.14	1.031 (1.123, 0.946)	0.49	1.113 (1.207, 1.026)	**0.001**
Sighthounds	5,111	0.739 (0.945, 0.578)	**0.016**	2.607 (6.622, 1.027)	**0.044**	1.482 (3.421, 0.642)	0.36	2.122 (4.872, 0.924)	0.076	1.45 (2.073, 1.014)	**0.042**	1.24 (1.784, 0.860)	0.25	1.177 (1.661, 0.834)	0.35

^*∗*^The reference groups were male, latitude ≥50°N, and summer.

The *p* values for statistically significant variables are in bold type.

**Table 4:** Severity of CHD by demographic parameters.

Parameter	CHD severity			% severity			*p* value
	Mild	Moderate	Severe	Mild	Moderate	Severe	
*Age (mos ± 1 sd)*	31.4 ± 8.4	32.0 ± 8.9	32.5 ± 9.3	—	—	—	<10^−6^
*Sex*							
Male	55,390	31,452	5,264	60.14	34.15	5.72	<10^−6^
Female	30,931	16,519	2,740	61.63	32.91	5.46	
*Laterality*							
Bilateral	51,085	36,754	7,537	53.56	38.54	7.90	<10^−6^
Unilateral	35,236	11,217	465	75.10	23.91	0.99	
*AKC group*							
Herding	17,011	8,906	1,723	61.54	32.22	6.23	<10^−6^
Hound	1,329	427	52	73.51	23.62	2.88	
Working	22,469	14,379	2,371	57.29	36.66	6.05	
Sporting	36,994	20,498	3,232	60.92	33.76	5.32	
Nonsporting	4,452	2,062	413	64.27	29.77	5.96	
Terrier	1,757	605	60	72.54	24.98	2.48	
Toy	1,622	800	105	64.19	31.66	4.16	
*FCI group*							
Sheep and cattle	17,387	9,106	1,763	61.53	32.23	6.24	<10^−6^
Pinscher schnauzer, molossoid, and Swiss Mtn/cattle dog	20,179	13,430	2,371	56.08	37.33	6.59	
Terrier	1,563	552	53	72.09	25.46	2.44	
Spitz and primitive	4,106	2,120	325	62.68	32.36	4.96	
Scent hounds	1,142	511	74	66.13	29.59	4.28	
Pointing dogs	4,981	2,496	290	64.13	32.14	3.73	
Retrievers, flushers, and water dogs	32,742	18,344	2,976	60.56	33.93	5.50	
Companion and toy dogs	3,710	1,178	126	73.99	23.49	2.51	
Sighthounds	181	85	3	67.29	31.60	1.12	
*Geographic group*							
<30°N	2,760	1,183	199	66.63	28.56	4.80	<10^−6^
30–39°N	41,628	22,781	3,711	61.11	33.44	5.45	
40–49°N	36,358	20,619	3,498	60.12	34.10	5.78	
≥50°N	5,335	3,235	580	58.31	35.36	6.34	
*Season of birth*							
Autumn	19,795	10,644	1,722	61.55	33.10	5.35	<10^−6^
Winter	21,512	12,101	1,960	60.47	34.02	5.51	
Spring	24,728	14,456	2,503	59.32	34.68	6.00	
Summer	20,286	10,770	1,819	61.71	32.76	5.53	

**(a):** 

Human DDH^*∗*^						Canine CHD^†^							
Race	Incidence per 1000 births	% M	% F	% bilateral	% unilateral	AKC groups	Prevalence	% bilateral	% unilateral	FCI groups	Prevalence	% bilateral	% unilateral
*Indigenous peoples*						*Sporting dogs*	15.03	66.03	33.87	*Sheep and cattle*	15.19	64.6	35.4
Native American	95.0	30	70	50	50	Retrievers	16.52	65.68	34.32	*Pinscher schnauzer, molossoid, and Swiss mountain/cattle dog*	20.43	69.3	30.7
Sami	40.0					Movers/flushers	12.71	68.7	31.3	*Terrier*	14.91	74.9	25.1
Aboriginal	3.7					Pointers/setters	12.13	65.96	34.04	*Spitz and primitive*	10.00	70.0	30.0
						Versatile sporting	8.42	60.16	39.84	*Scent hounds*	10.13	69.9	30.7
*Caucasian*						*Herding*	15.23	64.65	35.35	*Pointing dogs*	10.46	65.0	35.0
Eastern Europe	44.2	21	79	19	81	*Working*	18.04	68.85	31.15	*Retrievers, flushers, and water dogs*	10.91	66.2	33.8
Mediterranean Islands	14.3					*Nonsporting*	13.54	71.06	28.94	*Companion and toy dogs*	15.87	69.5	30.5
Australia/New Zealand	12.0	16	84	43	57	*Terrier*	12.59	73.86	66.14	*Sighthounds*	13.52	55.0	45.0
Western Europe	8.1	21	79	19	81	*Toy*	16.43	72.51	27.49				
United Kingdom	8.0					*Hound*	10.52	69.05	30.95				
Scandinavia	7.3	21	79	41	59	Scent hounds	12.97	71.0	29.0				
South America	4.6	24	76			Sighthounds	4.64	54.2	45.8				
North America	0.8	19	81	13	87								
*Indo-Mediterranean*	5.5	21	79	50	50								
*Indo-Malay*	0.4												
*Africans*													
North America	0.5												
Africa	0.1												

**(b):** 

Seasonal variation^‡^	*n*	%	Seasonal variation^†^	*n*	%
Single winter peak	16,425	70.3	Autumn (Sept-Nov)	20,276	2.93
Single summer peak	1,280	5.5	Winter (Dec–Feb)	312,202	45.27
Spring and autumn peaks	3,450	14.8	Spring (March–May)	229,925	33.23
No variation	2,205	9.4	Summer (June–Aug)	0	0
			No variation	128422	18.56

^*∗*^Data extracted from [5].

^‡^Data extracted from [6].

^†^Present study.
