# Supplementary material for: The Demographics of Canine Hip Dysplasia in the United States and Canada
Source: J Vet Med. 2017 Mar 12;2017:5723476. doi: 10.1155/2017/5723476 (PMC5366211; doi:10.1155/2017/5723476)
Supplement: Supplementary file 1 — The supplementary material in these gives detailed results of many of the analyses done, such as detailed prevalence or odds ratios for many specific breeds and groups. This is for the reader interested in more information. [file 5723476.f1.doc]

**Supplemental Table 1**

**Prevalence of CHD in dogs from the USA and Canada using the entire OFA data set**

| **Breed Name** | **n** | **(-) CHD** | **(+) CHD** | **% CHD (+)** |
| --- | --- | --- | --- | --- |
| Bulldog | 515 | 115 | 400 | 77.67 |
| Pug | 484 | 130 | 354 | 73.14 |
| Dogue de Bordeaux | 429 | 149 | 280 | 65.27 |
| Otterhound | 363 | 165 | 198 | 54.55 |
| Boerboel | 134 | 64 | 70 | 52.24 |
| Saint Bernard | 1241 | 594 | 647 | 52.14 |
| Neapolitan Mastiff | 132 | 64 | 68 | 51.52 |
| Basset Hound | 130 | 65 | 65 | 50.00 |
| Black Russian Terrier | 548 | 288 | 260 | 47.45 |
| Sussex Spaniel | 226 | 119 | 107 | 47.35 |
| Argentine Dogo | 182 | 101 | 81 | 44.51 |
| Cane Corso | 877 | 487 | 390 | 44.47 |
| Perro de Presa Canario | 168 | 98 | 70 | 41.67 |
| Norfolk Terrier | 240 | 142 | 98 | 40.83 |
| American Bulldog | 1446 | 870 | 576 | 39.83 |
| Boykin Spaniel | 2655 | 1641 | 1014 | 38.19 |
| Glen of Imaal Terrier | 135 | 84 | 51 | 37.78 |
| Spanish Water Dog | 114 | 73 | 41 | 35.96 |
| French Bulldog | 1111 | 732 | 379 | 34.11 |
| Fila Brasileiro | 473 | 320 | 153 | 32.35 |
| American Pit Bull Terrier | 2955 | 2078 | 877 | 29.68 |
| Lagotto Ramagnolo | 170 | 120 | 50 | 29.41 |
| Bloodhound | 2012 | 1422 | 590 | 29.32 |
| Newfoundland | 12142 | 8786 | 3356 | 27.64 |
| Bullmastiff | 4680 | 3418 | 1262 | 26.97 |
| English Shepherd | 331 | 247 | 84 | 25.38 |
| Cardigan Welsh Corgi | 1589 | 1191 | 398 | 25.05 |
| Shih Tzu | 384 | 292 | 92 | 23.96 |
| Louisiana Catahoula Leopard | 472 | 363 | 109 | 23.09 |
| Havana Silk Dog | 154 | 119 | 35 | 22.73 |
| Rottweiler | 75732 | 58579 | 17153 | 22.65 |
| Berger de Picard | 133 | 103 | 30 | 22.56 |
| Chesapeake Bay Retriever | 9393 | 7319 | 2074 | 22.08 |
| Mastiff | 9333 | 7277 | 2056 | 22.03 |
| Pembroke Welsh Corgi | 9364 | 7307 | 2057 | 21.97 |
| Golden Retriever | 99521 | 77894 | 21627 | 21.73 |
| German Shepherd Dog | 81749 | 64192 | 17557 | 21.48 |
| Chow Chow | 4013 | 3165 | 848 | 21.13 |
| Greater Swiss Mountain Dog | 2487 | 1973 | 514 | 20.67 |
| Chinook | 570 | 453 | 117 | 20.53 |
| Hybrid | 1537 | 1223 | 314 | 20.43 |
| Field Spaniel | 989 | 789 | 200 | 20.22 |
| Gordon Setter | 4268 | 3411 | 857 | 20.08 |
| Shiloh Shepherd | 749 | 599 | 150 | 20.03 |
| Polish Tatra Sheepdog | 140 | 112 | 28 | 20.00 |
| Norwegian Elkhound | 2445 | 1957 | 488 | 19.96 |
| Beagle | 733 | 587 | 146 | 19.92 |
| Pyrenean Shepherd | 127 | 102 | 25 | 19.69 |
| Affenpinscher | 285 | 231 | 54 | 18.95 |
| Staffordshire Bull Terrier | 515 | 418 | 97 | 18.83 |
| Kuvasz | 1374 | 1120 | 254 | 18.49 |
| Spinone Italiano | 1075 | 877 | 198 | 18.42 |
| Polish Lowland Sheepdog | 429 | 350 | 79 | 18.41 |
| Giant Schnauzer | 2966 | 2420 | 546 | 18.41 |
| Entlebucher | 298 | 246 | 52 | 17.45 |
| Curly-Coated Retriever | 918 | 758 | 160 | 17.43 |
| Tibetan Mastiff | 853 | 705 | 148 | 17.35 |
| Bernese Mountain Dog | 15653 | 12972 | 2681 | 17.13 |
| Old English Sheepdog | 5319 | 4411 | 908 | 17.07 |
| Australian Cattle Dog | 3189 | 2646 | 543 | 17.03 |
| Barbet | 102 | 85 | 17 | 16.67 |
| Labradoodle | 318 | 266 | 52 | 16.35 |
| Norwich Terrier | 792 | 663 | 129 | 16.29 |
| Bouvier des Flandres | 6549 | 5497 | 1052 | 16.06 |
| Black and Tan Coonhound | 456 | 383 | 73 | 16.01 |
| Leonberger | 1717 | 1444 | 273 | 15.90 |
| Briard | 1894 | 1596 | 298 | 15.73 |
| English Setter | 7786 | 6605 | 1181 | 15.17 |
| Brittany | 13426 | 11432 | 1994 | 14.85 |
| Cavalier King Charles Spaniel | 5755 | 4904 | 851 | 14.79 |
| Smooth Fox Terrier | 278 | 237 | 41 | 14.75 |
| Chinese Shar-Pei | 8215 | 7008 | 1207 | 14.69 |
| Harrier | 303 | 259 | 44 | 14.52 |
| Komondor | 613 | 525 | 88 | 14.36 |
| Iceland Sheepdog | 259 | 222 | 37 | 14.29 |
| Akita | 11936 | 10269 | 1667 | 13.97 |
| Portuguese Water Dog | 7105 | 6127 | 978 | 13.76 |
| Great Dane | 9670 | 8358 | 1312 | 13.57 |
| Beauceron | 394 | 341 | 53 | 13.45 |
| Boston Terrier | 186 | 161 | 25 | 13.44 |
| English Springer Spaniel | 11195 | 9691 | 1504 | 13.43 |
| Munsterlander | 179 | 155 | 24 | 13.41 |
| West Highland Terrier | 270 | 234 | 36 | 13.33 |
| Labrador Retriever | 181549 | 157458 | 24091 | 13.27 |
| Petit Basset Griffons Vendeen | 612 | 532 | 80 | 13.07 |
| Airedale Terrier | 4509 | 3936 | 573 | 12.71 |
| Boxer | 4496 | 3926 | 570 | 12.68 |
| Poodle | 18569 | 16239 | 2330 | 12.55 |
| Pudelpointer | 401 | 351 | 50 | 12.47 |
| Irish Water Spaniel | 1052 | 923 | 129 | 12.26 |
| Akbash Dog | 434 | 382 | 52 | 11.98 |
| Alaskan Malamute | 8582 | 7573 | 1009 | 11.76 |
| Swedish Vallhund | 222 | 196 | 26 | 11.71 |
| Havanese | 3067 | 2709 | 358 | 11.67 |
| Dutch Shepherd | 200 | 177 | 23 | 11.50 |
| Welsh Springer Spaniel | 1813 | 1605 | 208 | 11.47 |
| Anatolian Shepherd | 1641 | 1454 | 187 | 11.40 |
| Norwegian Buhund | 176 | 156 | 20 | 11.36 |
| Finnish Lapphund | 169 | 150 | 19 | 11.24 |
| Border Collie | 9479 | 8414 | 1065 | 11.24 |
| Samoyed | 10527 | 9346 | 1181 | 11.22 |
| Tibetan Spaniel | 250 | 222 | 28 | 11.20 |
| Irish Setter | 6423 | 5715 | 708 | 11.02 |
| Australian Kelpie | 111 | 99 | 12 | 10.81 |
| American Eskimo Dog | 822 | 736 | 86 | 10.46 |
| Puli | 1072 | 960 | 112 | 10.45 |
| Wirehaired Vizsla | 116 | 104 | 12 | 10.34 |
| Great Pyrenees | 4520 | 4078 | 442 | 9.78 |
| Cocker Spaniel | 11729 | 10603 | 1126 | 9.60 |
| Coton de Tulear | 712 | 645 | 67 | 9.41 |
| Weimaraner | 8591 | 7789 | 802 | 9.34 |
| Wirehaired Pointing Griffon | 1632 | 1483 | 149 | 9.13 |
| French Spaniel | 183 | 167 | 16 | 8.74 |
| Pointer | 1236 | 1128 | 108 | 8.74 |
| Standard Schnauzer | 3177 | 2910 | 267 | 8.40 |
| Schipperke | 347 | 318 | 29 | 8.36 |
| Small Musterlander | 144 | 132 | 12 | 8.33 |
| German Wirehaired Pointer | 3713 | 3406 | 307 | 8.27 |
| American Water Spaniel | 571 | 524 | 47 | 8.23 |
| Miniature Australian Shepherd | 1649 | 1514 | 135 | 8.19 |
| Bichon Frise | 2835 | 2608 | 227 | 8.01 |
| Vizsla | 10437 | 9668 | 769 | 7.37 |
| Nova Scotia Duck Tolling Retriever | 1771 | 1644 | 127 | 7.17 |
| Lhasa Apso | 309 | 287 | 22 | 7.12 |
| Afghan Hound | 2792 | 2595 | 197 | 7.06 |
| Keeshond | 3509 | 3267 | 242 | 6.90 |
| Tibetan Terrier | 2923 | 2726 | 197 | 6.74 |
| Doberman Pinscher | 11399 | 10633 | 766 | 6.72 |
| Finnish Spitz | 262 | 245 | 17 | 6.49 |
| English Cocker Spaniel | 5161 | 4840 | 321 | 6.22 |
| Kerry Blue Terrier | 1207 | 1133 | 74 | 6.13 |
| Bearded Collie | 3400 | 3192 | 208 | 6.12 |
| Shiba Inu | 2835 | 2662 | 173 | 6.10 |
| Australian Shepherd | 27187 | 25539 | 1648 | 6.06 |
| Belgian Malinois | 2479 | 2339 | 140 | 5.65 |
| North American Shepherd | 285 | 269 | 16 | 5.61 |
| Soft Coated Wheaten Terrier | 4842 | 4576 | 266 | 5.49 |
| Hovawart | 130 | 123 | 7 | 5.38 |
| Irish Red and White Setter | 216 | 205 | 11 | 5.09 |
| Irish Wolfhound | 1286 | 1222 | 64 | 4.98 |
| Shetland Sheepdog | 15552 | 14806 | 746 | 4.80 |
| Flat-Coated Retriever | 4403 | 4194 | 209 | 4.75 |
| German Shorthaired Pointer | 10841 | 10356 | 485 | 4.47 |
| Rhodesian Ridgeback | 8869 | 8478 | 391 | 4.41 |
| Border Terrier | 2366 | 2266 | 100 | 4.23 |
| Dalmatian | 2889 | 2771 | 118 | 4.08 |
| Belgian Tervuren | 4341 | 4167 | 174 | 4.01 |
| Basenji | 2121 | 2045 | 76 | 3.58 |
| Belgian Sheepdog | 2810 | 2715 | 95 | 3.38 |
| Australian Terrier | 149 | 144 | 5 | 3.36 |
| Collie | 1969 | 1903 | 66 | 3.35 |
| Rat Terrier | 421 | 407 | 14 | 3.33 |
| Eurasier | 102 | 99 | 3 | 2.94 |
| Canaan | 323 | 314 | 9 | 2.79 |
| Toy Australian Shepherd | 116 | 113 | 3 | 2.59 |
| Ibizan Hound | 241 | 236 | 5 | 2.07 |
| Siberian Husky | 9891 | 9689 | 202 | 2.04 |
| German Pinscher | 358 | 351 | 7 | 1.96 |
| Pharaoh Hound | 419 | 411 | 8 | 1.91 |
| Borzoi | 409 | 405 | 4 | 0.98 |
| Greyhound | 230 | 228 | 2 | 0.87 |
| Whippet | 124 | 123 | 1 | 0.81 |
| Italian Greyhound | 173 | 173 | 0 | 0.00 |

CHD = canine hip dysplasia, - = no hip dysplasia, + = hip dysplasia

**Supplemental Table 2**

**Odds ratios of CHD within AKC/FCI subgroups by sex, season of birth and latitude***

|  |  |  | **Sex** | | **Latitude** | | | | | | **Season of birth** | | | | | |
| --- | --- | --- | --- | --- | --- | --- | --- | --- | --- | --- | --- | --- | --- | --- | --- | --- |
|  |  |  | **Female** |  | **<30° N** |  | **30-39° N** |  | **40-49° N** |  | **Autumn** |  | **Winter** |  | **Spring** |  |
| **AKC Subgroups** | **% CHD** | **n** | **OR (95% CI)** | **p value** | **OR (95% CI)** | **p value** | **OR (95% CI)** | **p value** | **OR (95% CI)** | **p value** | **OR (95% CI)** | **p value** | **OR (95% CI)** | **p value** | **OR (95% CI)** | **p value** |
| **Scenthounds** | 12.97 | 15,930 | 1.052 (1.157, 0.956) | 0.30 | 1.138 (1.594, 0.812) | 0.81 | 1.049 (1.404, 0.783) | 0.78 | 1.156 (1.550, 0.862) | 0.86 | 0.979 (1.118, 0.856) | 0.75 | 1.088 (1.237, 0.956) | 0.20 | 0.889 (1.007, 0.783) | 0.065 |
| **Sighthounds** | 4.64 | 7,691 | 0.829 (1.026, 0.670) | 0.085 | 2.597 (6.965, 1.112) | 0.027 | 1.322 (2.857, 0.612) | 0.48 | 1.934 (4.165, 0.898) | 0.092 | 1.337 (1.849, 0.967) | 0.078 | 1.87 (1.478, 0.799) | 0.60 | 1.147 (1.595, 0.825) | 0.42 |
| **Swiss Mountain Dogs** | 17.61 | 18,098 | 0.994 (1.076, 0.919) | 0.89 | 2.5 (3.481, 1.796) | **<10^-6^** | 1.742 (2.142, 1.417) | **<10^-6^** | 1.81 (2.219, 1.476) | **<10^-6^** | 1.05 (1.176, 0.938) | 0.40 | 1.127 (1.258, 1.009) | 0.035 | 1.14 (1.270, 1.024) | **0.017** |
| **Mastiffs** | 24.95 | 27,363 | 1.055 (1.117, 0.997) | 0.063 | 2.379 (2.977, 1.901) | **<10^-6^** | 1.374 (1.669, 1.131) | **0.0014** | 1.614 (1.960, 1.330) | **0.000001** | 0.949 (1.016, 0.867 | 0.12 | 1.009 (1.090, 0.933) | 0.83 | 1.176 (1.272, 1.086) | **0.00006** |
| **Pointers/Setters** | 12.13 | 42,187 | 0.987 (1.044, 0.930) | 0.68 | 1.464 (1.865, 1.149) | **0.002** | 1.094 (1.322, 0.905) | 0.36 | 1.158 (1.397, 0.960) | 0.12 | 1.097 (1.199, 1.004) | **0.041** | 1.215 (1.323, 1.117) | **0.000007** | 1.179 (1.272, 1.092) | **0.00002** |
| **Movers/Flushers** | 12.71 | 38,282 | 0.899 (0.957, 0.846) | **0.0008** | 2.027 (2.528, 1.626) | **<10^-6^** | 1.785 (2.135, 1.492) | **<10^-6^** | 1.4 (1.675, 1.170) | **<10^-6^** | 1.047 (1.145, 0.957) | 0.32 | 1.1 (1.201, 1.008) | **0.033** | 1.186 (1.287, 1.092) | **0.00005** |
| **Retrievers** | 16.52 | 282,982 | 1.022 (1.044, 1.001) | **0.041** | 1.977 (2.228, 1.755) | **<10^-6^** | 1.315 (1.471, 1.176) | **<10^-6^** | 1.571 (1.756, 1.404) | **<10^-6^** | 1.048 (1.080, 1.018) | **0.0018** | 1.166 (1.200, 1.133) | **<10^-6^** | 1.199 (1.232, 1.166) | **<10^-6^** |
| **Versatile Sporting** | 8.42 | 20,081 | 1.097 (1.218, 0.987) | 0.085 | 1.829 (3.584, 0.933) | 0.079 | 1.744 (3.307, 0.920) | 0.089 | 1.972 (3.737, 1.041) | **0.037** | 1.361 (1.582, 1.171) | **0.00006** | 1.471 (1.705, 1.270) | **<10^-6^** | 1.275 (1.464, 1.110) | **0.00058** |
| **Bulldogs** | 44.21 | 3,092 | 0.98 (1.130, 0.849) | 0.78 | 0.969 (1.684, 0.557) | 0.91 | 1.115 (1.822, 0.682) | 0.66 | 1.267 (2.078, 0.773) | 0.35 | 0.935 (1.142, 0.765) | 0.51 | 1.166 (1.433, 0.949) | 0.14 | 1.136 (1.384, 0.932) | 0.21 |
| **FCI Subgroups** |  |  |  |  |  |  |  |  |  |  |  |  |  |  |  |  |
| **Sheep** | 15.12 | 9,743 | 0.926 (1.034, 0.830) | 0.17 | 1.361 (1.854, 0.999) | 0.051 | 1.095 (1.357, 0.884) | 0.41 | 0.99 (1.233, 0.794) | 0.93 | 0.857 (1.006, 0.731) | 0.059 | 0.938 (1.096, 0.804) | 0.42 | 1.047 (1.209, 0.907) | 0.53 |
| **Cattle** | 16.36 | 9,743 | 0.926 (1.034, 0.830) | 0.17 | 1.361 (1.854, 0.999) | 0.051 | 1.095 (1.357, 0.884) | 0.41 | 0.99 (1.233, 0.794) | 0.93 | 0.857 (1.006, 0.731) | 0.059 | 0.938 (1.096, 0.804) | 0.42 | 1.047 (1.209, 0.907) | 0.53 |
| **Pinscher** | 6.54 | 11,816 | 0.957 (1.100, 0.815) | 0.48 | 1.855 (2.967, 1.160) | **0.0098** | 1.52 (2.304, 1.003) | **0.048** | 1.425 (2.163, 0.939) | 0.096 | 1.171 (1.455, 0.942) | 0.15 | 1.143 (1.407, 0.929) | 0.21 | 1.008 (1.236, 0.822) | 0.94 |
| **Schnauzer** | 13.15 | 6,147 | 1.314 (1.53, 1.122) | **0.0007** | 2.611 (4.494, 1.517) | **0.0005** | 1.576 (2.529, 0.982) | **0.059** | 1.474 (2.364, 0.919) | 0.11 | 0.995 (1.233, 0.803) | 0.96 | 1.088 (1.344, 0.881) | 0.43 | 1.003 (1.227, 0.820) | 0.98 |
| **Mastiffs** | 22.04 | 116,164 | 1.087 (1.119, 1.057) | **<10^-6^** | 2.097 (2.307, 1.907) | **<10^-6^** | 1.458 (1.590, 1.338) | **<10^-6^** | 1.458 (1.590, 1.338) | **<10^-6^** | 0.994 (1.035, 0.955) | 0.77 | 1.084 (1.129, 1.041) | **0.00009** | 1.215 (1.263, 1.168) | **<10^-6^** |
| **Mountain Dogs** | 22.86 | 22,322 | 1.096 (1.169, 1.027) | **0.006** | 2.822 (3.611, 2.205) | **<10^-6^** | 1.36 (1.601, 1.155) | **0.0002** | 1.74 (2.42, 1.483) | **<10^-6^** | 0.894 (0.980, 0.816) | **0.017** | 0.951 (1.040, 0.869) | 0.27 | 1.101 (1.205, 1.005) | **0.039** |
| **Swiss Mtn Dog** | 17.63 | 18,375 | 1.007 (1.088, 0.931) | 0.87 | 2.505 (3.479, 1.804) | **<10^-6^** | 1.706 (2.096, 1.389) | **<10^-6^** | 1.769 (2.167, 1.444) | **<10^-6^** | 1.053 (1.178, 0.941) | 0.37 | 1.126 (1.257, 1.009) | 0.035 | 1.153 (1.282, 1.036) | **0.009** |
| **Large/Medium Terrier** | 9.86 | 8,736 | 1.068 (1.241, 0.919) | 0.39 | 1.249 (2.068, 0.754) | 0.39 | 1.204 (1.734, .0836) | 0.32 | 1.66 (2.370, 1.162) | **0.005** | 1.076 (1.324, 0.875) | 0.49 | 1.129 (1.387, 0.919) | 0.25 | 1.224 (1.480, 1.011) | **0.038** |
| **Small Terrier** | 14.80 | 2,182 | 1.215 (1.558, 0.948) | 0.12 | 1.957 (5.790, 0.662) | 0.22 | 1.495 (3.318, 0.674) | 0.32 | 2.215 (4.902, 1.000) | 0.049 | 0.802 (1.138, 0.566) | 0.22 | 0.923 (1.293, 0.659) | 0.64 | 0.934 (1.293, 0.675) | 0.68 |
| **Bull Terrier** | 27.60 | 3,557 | 0.93 (1.9085, 0.798) | 0.36 | 1.061 (1.700, 0.662) | 0.81 | 0.647 (0.993, 0.422) | 0.047 | 0.775 (1.196, 0.502) | 0.25 | 1.073 (1.329, 0.867) | 0.52 | 1.236 (1.518, 1.006) | 0.044 | 1.103 (1.350, 0.901) | 0.34 |
| **Sledge Dog** | 8.52 | 29,482 | 1.034 (1.126, 0.950) | 0.44 | 1.559 (2.093, 1.161) | **0.003** | 1.136 (1.367, 0.944) | 0.18 | 1.207 (1.448, 1.005) | 0.044 | 1.005 (1.129, 0.894) | 0.94 | 1.065 (1.197, 0.948) | 0.29 | 0.993 (1.115, 0.885) | 0.91 |
| **Hunting Dog** | 18.52 | 2,768 | 0.945 (1.149, 0.777) | 0.57 | 0.0847 (2.821, 0.254) | 0.79 | 0.843 (1.464, 0.486) | 0.54 | 0.054 (1.652, 0.551) | 0.87 | 0.918 (1.205, 0.699) | 0.54 | 1.065 (1.387, 0.818) | 0.64 | 0.902 (1.183, 0.688) | 0.46 |
| **Watch Dog** | 6.59 | 5,664 | 1.037 (1.305, 0.824) | 0.76 | 1.412 (3.181, 0.627) | 0.41 | 1.295 (2.099, 0.799) | 0.29 | 1.502 (2.405, 0.938) | 0.09 | 1.215 (1.644, 0.897) | 0.21 | 1.148 (1.573, 0.838) | 0.39 | 1.101 (1.491, 0.814) | 0.53 |
| **European Spitz** | 7.64 | 4,413 | 1.253 (1.580, 0.993) | 0.057 | 0.552 (1.625, 0.187) | 0.28 | 1.377 (2.582, 0.734) | 0.32 | 1.362 (2.565, 0.724) | 0.34 | 0.851 (1.160, 0.624) | 0.31 | 0.837 (1.154, 0.607) | 0.28 | 0.953 (1.294, 0.703) | 0.76 |
| **Asian Spitz** | 14.23 | 18,829 | 0.964 (1.049, 0.886) | 0.40 | 1.725 (2.190, 1.358) | **0.000008** | 1.004 (1.207, 0.835) | 0.97 | 1.099 (1.324, 0.912) | 0.33 | 0.931 (1.039, 0.834) | 0.20 | 1.083 (1.219, 0.962) | 0.19 | 0.905 (1.032, 0.794) | 0.13 |
| **Primitive** | 3.29 | 2,879 | 1.084 (1.647, 0.714) | 0.71 | 4.399 (37.43, 0.517) | 0.18 | 2.134 (15.77, 0.289) | 0.46 | 3.149 (23.24, 0.427) | 0.26 | 1.965 (4.581, 0.842) | 0.12 | 1.761 (3907, 0.794) | 0.16 | 1.044 (3.149, 0.346) | 0.94 |
| **Large Scenthound** | 29.87 | 2,912 | 1.456 (1.718, 1.234) | **0.000008** | 0.787 (1.323, 0.467) | 0.37 | 0.702 (1.085, 0.454) | 0.11 | 0.815 (1.264, 0.525) | 0.36 | 0.967 (1.220, 0.767) | 0.78 | 1.058 (1.322, 0.846) | 0.62 | 0.903 (1.118, 0.730) | 0.35 |
| **Small Scenthound** | 19.80 | 1,504 | 0.834 (1.080, 0.645) | 0.17 | 1.008 (2.070, 0.491) | 0.98 | 1.55 (2.828, 0.850) | 0.15 | 1.21 (2.221, 0.659) | 0.53 | 0.907 (1.300, 0.634) | 0.60 | 0.988 (1.407, 0.694) | 0.95 | 0.78 (1.104, 0.551) | 0.16 |
| **Leash Hound** | 4.33 | 11,747 | 1.159 (1.399, 0.961) | 0.12 | 1.829 (3.226, 1.037) | **0.037** | 1.091 (1.826, 0.652) | 0.74 | 0.836 (1.415, 0.494) | 0.50 | 1.163 (1.514, 0.893) | 0.26 | 1.193 (1.551, 0.919) | 0.19 | 1.184 (1.513, 0.926) | 0.18 |
| **Continental Pointers** | 7.16 | 34,253 | 1.031 (1.124, 0.947) | 0.48 | 2.627 (4.023, 1.716) | **0.000009** | 1.08 (3.071, 1.409) | **0.00023** | 2.355 (3.470, 1.598) | **0.00002** | 1.377 (1.556, 1.218) | **<10^-6^** | 1.442 (1.628, 1.277) | **<10^-6^** | 1.198 (1.342, 1.070) | **0.0017** |
| **Pointer Spaniels** | 14.73 | 14,105 | 1.158 (1.276, 1.051) | **0.0031** | 2.181 (3.324, 1.431) | **0.0029** | 1.369 (1.943, 0.964) | 0.079 | 1.158 (1.278, 1.051) | 0.07 | 1.166 (1.343, 1.012) | **0.033** | 1.146 (1.318, 0.996) | 0.056 | 1.29 (1.453, 1.145) | **0.00003** |
| **Pointer Griffon** | 12.72 | 2,729 | 1.212 (1.537, 0.956) | 0.11 | 1.64 (6.934, 0.388) | 0.50 | 2.921 (8.181, 1.043) | **0.041** | 2.206 (6.126, 0.795) | 0.13 | 1.207 (1.757, 0.829) | 0.33 | 1.588 (2.200, 1.147) | **0.0054** | 1.19 (1.632, 0.869) | 0.28 |
| **British Pointer** | 8.74 | 1,234 | 0.875 (1.303, 0.588) | 0.51 | 2.861 (26.88, 0.305) | 0.36 | 3.156 (23.54, 0.423) | 0.26 | 3.08 (23.12, 0.410) | 0.27 | 0.947 (1.651, 0.543) | 0.85 | 0.753 (1.273, 0.445) | 0.29 | 0.569 (0.984, 0.329) | **0.044** |
| **British Setter** | 14.77 | 18,640 | 0.908 (0.985, 0.836) | **0.021** | 1.087 (1.466, 0.896) | 0.58 | 0.907 (1.128, 0.729) | 0.38 | 0.998 (1.237, 0.805) | 0.98 | 0.963 (1.092, 0.849) | 0.55 | 1.192 (1.337, 1.062) | **0.0028** | 1.182 (1.315, 1.063) | **0.002** |
| **Retrievers** | 16.23 | 296,777 | 1.023 (1.945, 1.002) | **0.031** | 2.364 (2.547, 2.194) | **<10^-6^** | 1.573 (1.674, 1.478) | **<10^-6^** | 1.848 (1.966, .738) | **<10^-6^** | 1.052 (1.083, 1.022) | **0.0006** | 1.163 (1.197, 1.130) | **<10^-6^** | 1.195 (1.229, 1.163) | **<10^-6^** |
| **Flushers** | 13.32 | 33,772 | 0.902 (0.962, 0.846) | **0.0017** | 2.152 (2.732, 1.696) | **<10^-6^** | 1.97 (2.389, 1.624) | **<10^-6^** | 1.521 (1.846, 1.254) | **<10^-6^** | 1.055 (1.158, 0.962) | 0.26 | 1.093 (1.198, 0.998) | 0.056 | 1.206 (1.314, 1.106) | **0.00002** |
| **Water Dogs** | 13.85 | 9,102 | 0.953 (1.077, 0.844) | 0.44 | 1.217 (1.847, 0.802) | 0.36 | 0.84 (1.130, 0.625) | 0.25 | 0.852 (1.137, 0.638) | 0.28 | 0.661 (0.799, 0.547) | **0.00002** | 0.906 (1.068, 0.769) | 0.24 | 0.914 (1.069, 0.782) | 0.26 |
| **Bichon** | 10.16 | 6,791 | 1.386 (1.650, 1.64) | **0.0002** | 1.326 (2.151, 0.817) | 0.25 | 1.056 (1.622, 0.688) | 0.80 | 1.075 (1.654, 0.698) | 0.74 | 0.803 (1.016, 0.634) | 0.068 | 1.145 (1.425, 0.920) | 0.23 | 1.064 (1.321, 0.857) | 0.58 |
| **Poodle** | 12.55 | 18,519 | 0.981 (1.075, 0.894) | 0.68 | 1.482 (1.930, 1.139) | 0.0035 | 1.21 (1.489, 0.983) | 0.073 | 1.289 (1.586, 1.046) | **0.017** | 0.886 (1.004, 0.781) | 0.058 | 0.912 (1.035, 0.804) | 0.15 | 1.042 (1.169, 0.928) | 0.49 |
| **Tibetan** | 8.77 | 3,864 | 0.994 (1.251, 0.789) | 0.96 | 2.173 (5.127, 0.921) | 0.076 | 1.429 (3.128, 0.653) | 0.37 | 1.795 (3.912, 0.824) | 0.14 | 0.865 (1.201, 0.623) | 0.39 | 1.029 (1.417, 0.747) | 0.86 | 1.269 (1.713, 0.940) | 0.12 |
| **English Toy Spaniel** | 0.17 | 5,768 | 0.862 (1.003, 0.742) | 0.054 | 1.734 (3.689, 0.893) | 0.10 | 1.624 (2.754, 0.958) | 0.072 | 1.27 (2.161, 0.747) | 0.38 | 1.038 (1.280, 0.842) | 0.72 | 1.139 (1.405, 0.923) | 0.23 | 1.18 (1.451, 0.959) | 0.12 |
| **Long Haired/Fringed Hounds** | 6.13 | 3,286 | 0.627 (0.836, 0.471) | **0.0015** | 3.114 (13.935, 0.696) | 0.14 | 2.311 (9.577, 0.557) | 0.25 | 3.544 (14.632, 0.859) | 0.081 | 1.421 (2.315, 0.945) | 0.092 | 1.1 (1.691, 0.716) | 0.66 | 1.071 (1.588, 0.722) | 0.73 |
| **Rough Haired Hounds** | 0.56 | 1,291 | 1.413 (2.499, 0.799) | 0.23 | 2.741 (11.84, 0.635) | 0.18 | 0.724 (2.174, 0.241) | 0.56 | 0.961 (2.802, 0.330) | 0.94 | 1.331 (2.984, 0.594) | 0.49 | 1.662 (3.477, 0.795) | 0.18 | 1.4 (2.940, 0.665) | 0.38 |

* The reference groups were male, latitude > 50° N, and summer.

The p values for statistically significant variables are in bold type.

**Supplemental Table 3**

**Odds ratios of CHD for the 25 most common breeds by sex, season of birth and latitude***

|  |  |  | **Sex** | | **Latitude** | | | | | | **Season of birth** | | | | | |
| --- | --- | --- | --- | --- | --- | --- | --- | --- | --- | --- | --- | --- | --- | --- | --- | --- |
|  |  |  | **Female** |  | **<30° N** |  | **30-39° N** |  | **40-49° N** |  | **Autumn** |  | **Winter** |  | **Spring** |  |
| **Breed** | **% CHD** | **n** | **OR (95%CI)** | **p value** | **OR (95%CI)** | **p value** | **OR (95%CI)** | **p value** | **OR (95%CI)** | **p value** | **OR (95%CI)** | **p value** | **OR (95%CI)** | **p value** | **OR (95%CI)** | **p value** |
| **Labrador Retriever** | 13.27 | 181,229 | 0.991 (1.020, 0.963) | 0.55 | 2.141 (1.385, 1.922) | **<10^-6^** | 1.555 (1.705, 1.417) | **<10^-6^** | 1.793 (1.967, 1.636) | **<10^-6^** | 1.098 (1.144, 1.054) | **0.000007** | 1.279 (1.331, 1.229) | **<10^-6^** | 1.255 (1.304, 1.208) | **<10^-6^** |
| **Golden Retriever** | 21.73 | 99,111 | 1.031 (1.065, 0.998) | 0.063 | 3.172 (3.553, 2.832) | **<10^-6^** | 1.726 (1.893, 1.574) | **<10^-6^** | 2.025 (2.219, 1.848) | **<10^-6^** | 0.989 (1.034, 0.945) | 0.64 | 1.022 (1.068, 0.978) | 0.33 | 1.161 (1.210, 1.113) | **<10^-6^** |
| **German Shepherd** | 21.48 | 81,443 | 1.267 (1.312, 1.224) | **<10^-6^** | 2.33 (2.605, 2.083) | **<10^-6^** | 1.481 (1.631, 1.346) | **<10^-6^** | 1.668 (1.836, 1.515) | **<10^-6^** | 1.017 (1.069, 0.967) | 0.51 | 1.26 (1.321, 1.201) | **<10^-6^** | 1.166 (1.222, 1.113) | **<10^-6^** |
| **Rottweiler** | 22.65 | 75,450 | 1.154 (1.195, 1.114) | **<10^-6^** | 1.986 (2.235, 1.764) | **<10^-6^** | 1.395 (1.556, 1.251) | **<10^-6^** | 1.489 (1.662, 1.333) | **<10^-6^** | 0.972 (1.023, 0.934) | 0.28 | 1.072 (1.127, 1.020) | **0.0065** | 1.266 (1.329, 1.207) | **<10^-6^** |
| **Australian Shepherd** | 6.06 | 27,163 | 1.058 (1.174, 0.953) | 0.29 | 2.719 (4.272, 1.730) | **0.00001** | 1.984 (3.000, 1.314) | **0.0011** | 1.917 (2.905, 1.266) | **0.002** | 0.92 (1.070, 0.791) | 0.28 | 1.081 (1.247, 0.937) | 0.28 | 1.117 (1.282, 0974) | 0.11 |
| **Poodle** | 12.55 | 18,519 | 0.981 (1.075, 0.894) | 0.68 | 1.482 (1.930, 1.139) | **0.0035** | 1.21 (1.489, 0.983) | 0.073 | 1.288 (1.586, 1.046) | **0.017** | 0.886 (1.004, 0.781) | 0.058 | 0.912 (1.035, 0.804) | 0.15 | 1.042 (1.169, 0.928) | 0.49 |
| **Bernese Mountain Dog** | 17.13 | 15,590 | 1.048 (1.142, 0.962) | 0.29 | 2.442 (3.629, 1.643) | **0.00001** | 1.661 (2.050, 1.345) | **0.000002** | 1.661 (2.043, 1.351) | **0.000002** | 1.049 (1.188, 0.927) | 0.45 | 1.167 (1.318, 1.034) | **0.013** | 1.164 (1.309, 1.035) | **0.011** |
| **Shetland Sheepdog** | 4.80 | 15,518 | 1.072 (1.255, 0.916) | 0.38 | 1.809 (3.022, 1.082) | **0.024** | 1.796 (2.763, 1.168) | **0.0078** | 1.904 (2.919, 1.242) | **0.0031** | 1.318 (1.639, 1.059) | **0.013** | 1.376 (1.690, 1.120) | **0.0024** | 1.086 (1.334, 0.884) | 0.43 |
| **Brittany** | 14.85 | 13,399 | 1.162 (1.283, 1.052) | **0.003** | 2.426 (3.791, 1.552) | **0.0001** | 1.538 (2.247, 1.052) | **0.026** | 1.56 (12.275, 1.070) | **0.021** | 1.162 (1.342, 1.007) | **0.04** | 1.115 (1.287, 0.967) | 0.14 | 1.279 (1.444, 1.113) | **0.00007** |
| **Newfoundland** | 27.64 | 12,085 | 1.065 (1.158, 0.979) | 0.14 | 5.844 (8.123, 4.204) | **<10^-6^** | 1.982 (2.450, 1.603) | **<10^-6^** | 2.078 (2.553, 1.691) | **<10^-6^** | 0.857 (0.961, 0.765) | **0.008** | 1.008 (1.130, 0.900) | 0.90 | 1.267 (1.421, 1.128) | **0.000063** |
| **Akita** | 13.97 | 11,869 | 1.015 (1.132, 0.910) | 0.79 | 1.68 (2.245, 1.257) | **0.00046** | 0.881 (1.111, 0.698) | 0.29 | 1.03 (1.305, 0.813) | 0.81 | 0.997 (1.150, 0.865) | 0.97 | 1.136 (1.323, 0.975) | 0.10 | 0.922 (1.034, 0.777) | 0.35 |
| **Cocker Spaniel** | 9.60 | 11,697 | 0.834 (0.45, 0.735) | **0.0044** | 1.675 (2.568, 1.093) | **0.018** | 1.292 (1.874, 0.891) | 0.18 | 1.333 (1.933, 0.920) | 0.13 | 1.01 (1.213, 0.840) | 0.92 | 1.071 (1.280, 0.896) | 0.45 | 1.146 (1.360, 0.966) | 0.12 |
| **Doberman Pinscher** | 6.72 | 11,372 | 0.937 (1.090, 0.806) | 0.40 | 1.898 (3.060, 1.178) | **0.0085** | 1.603 (2.448, 1.049) | **0.029** | 1.463 (2.238, 0.956) | 0.079 | 1.19 (1.481, 0.956) | 0.12 | 1.129 (1.391, 0.917) | 0.25 | 0.993 (1.220, 0.809) | 0.95 |
| **English Springer Spaniel** | 13.43 | 11,159 | 0.932 (1.043, 0.832) | 0.22 | 2.531 (3.734, 1.716) | **0.000003** | 1.522 (2.018, 1.148) | **0.0035** | 1.587 (2.090, 1.206) | **0.0001** | 1.133 (1333, 0.965) | 0.13 | 1.13 (1.326, 0.964) | 0.13 | 1.22 (1.413, 1.054) | **0.008** |
| **German Shorthaired Pointer** | 4.47 | 10,839 | 0.926 (1.117, 0.768) | 0.42 | 3.953 (29.71, 0.526) | 0.18 | 1.64 (12.00, 0.224) | 0.63 | 2.151 (15.69, 0.295) | 0.45 | 1.415 (1.843, 1.086) | **0.010** | 1.416 (1.855, 1.081) | **0.011** | 1.102 (1.414, 0.859) | 0.45 |
| **Samoyed** | 11.22 | 10,500 | 0.981 (1.111, 0.866) | 0.76 | 1.564 (2.302, 1.063) | **0.023** | 0.928 (1.209, 0.713) | 0.58 | 0.988 (1.281, 0.762) | 0.93 | 0.96 (1.140, 0.808) | 0.64 | 0.96 (1.139, 0.809) | 0.64 | 1.032 (1.224, 0.871) | 0.71 |
| **Viszla** | 7.37 | 10,422 | 1.064 (1.241, 0.912) | 0.43 | 1.059 (2.466, 0.455) | 0.89 | 1.329 (2.871, 0.615) | 0.47 | 1.616 (3.481, 0.750) | 0.22 | 1.464 (1.811, 1.184) | **0.0004** | 0.272 (1.588, 1.027) | **0.028** | 1.12 (1.371, 0.915) | 0.27 |
| **Siberian Husky** | 2.04 | 9,854 | 1.316 (1.791, 0.966) | 0.081 | 4.739 (18.00, 1.247) | **0.022** | 3.665 (11.64, 1.154) | **0.028** | 4.08 (12.90, 1.290) | **0.017** | 1.632 (2.516, 1.067) | **0.024** | 1.495 (1.326, 0.961) | 0.074 | 1.436 (2.216, 0.930) | 0.10 |
| **Great Dane** | 13.57 | 9,647 | 0.999 (1.128, 0.885) | 0.99 | 4.272 (6.505, 2.806) | **<10^-6^** | 2.146 (3.137, 1.468) | **<10^-6^** | 2.681 (3.919, 1.833) | **0.00008** | 1.024 (1.218, 0.861) | 0.79 | 1.128 (1.336, 0.953) | 0.16 | 1.095 (1.286, 0.933) | 0.27 |
| **Border Collie** | 11.24 | 9,471 | 0.829 (0.943, 0.730) | **0.0043** | 1.736 (2.781, 1.084) | **0.022** | 1.049 (1.567, 0.703) | 0.82 | 1.235 (1.847, 0.826) | 0.30 | 1.042 (1.265, 0.860) | 0.68 | 1.007 (1.210, 0.838) | 0.94 | 1.127 (1.345, 0.945) | 0.18 |
| **Chespeake Bay Retriever** | 22.08 | 9,376 | 1.076 (1.192, 0.971) | 0.16 | 1.601 (2.361, 1.086) | **0.018** | 1.324 (1.770, 0.990) | 0.058 | 1.29 (1.721, 0.967) | 0.083 | 1.179 (1.367, 1.0-18) | **0.028** | 1.364 (1.572, 1.184) | **0.000017** | 1.463 (1.676, 1.277) | **<10^-6^** |
| **Pembroke Welsh Corgi** | 21.97 | 9,345 | 1.133 (1.260, 1.018) | **0.022** | 1.392 (2.022, 0.958) | 0.083 | 1.258 (1.720, 0.920) | 0.15 | 1.127 (1.543, 0.823) | 0.46 | 0.774 (0.895, 0.670) | **0.0005** | 0.967 (1.114, 0.840) | 0.65 | 1.206 (1.379, 1.055) | **0.0061** |
| **Mastiff** | 22.03 | 9,319 | 1.107 (1.226, 0.999) | 0.052 | 2.538 (3.904, 1.650) | **0.0002** | 1.9 (2.824, 1.279) | **0.0015** | 1.876 (2.707, 1.260) | **0.0019** | 0.999 (1.150, 0.867) | 0.098 | 1.061 (1.219, 0.924) | 0.40 | 1.135 (1.305, 0.987) | 0.076 |
| **Rhodesian Ridgeback** | 4.41 | 8,865 | 1.128 (1.398, 0.911) | 0.27 | 3.495 (8.263, 1.478) | **0.0044** | 2.067 (4.697, 0.909) | 0.083 | 1.632 (3.743, 0.711) | 0.25 | 1.0902 (1.464, 0.814) | 0.56 | 1.134 (1.515, 0.849) | 0.39 | 0.974 (1.287, 0.737) | 0.85 |
| **Weimaraner** | 9.34 | 8,583 | 1.156 (1.351, 0.989) | 0.068 | 3.326 (6.472, 1.710) | **0.0004** | 2.512 (4.637, 1.361) | **0.0032** | 2.693 (4.967, 1.460) | **0.0015** | 1.268 (1.586, 1.014) | **0.038** | 1.622 (2.015, 1.306) | **0.000012** | 1.446 (1.776, 1.178) | **0.0043** |

* The reference groups were male, latitude > 50° N, and summer.

The p values for statistically significant variables are in bold type.

**Supplemental Table 4**

**Odds ratios of CHD for all dogs with an n > 1000 regardless of CHD prevalence, and those with both an n > 100 and a CHD prevalence of > 15%**

|  |  |  | **Sex** |  | **Latitude** |  |  |  |  |  | **Season of birth** |  |  |  |  |  |
| --- | --- | --- | --- | --- | --- | --- | --- | --- | --- | --- | --- | --- | --- | --- | --- | --- |
| **All dogs with n > 1000** |  |  | **Female** |  | **<30° N** |  | **30-39° N** |  | **40-49° N** |  | **Autumn** |  | **Winter** |  | **Spring** |  |
| **By Individual Breeds** | **% CHD** | **n** | **OR (95%CI)** | **p value** | **OR (95%CI)** | **p value** | **OR (95%CI)** | **p value** | **OR (95%CI)** | **p value** | **OR (95%CI)** | **p value** | **OR (95%CI)** | **p value** | **OR (95%CI)** | **p value** |
| **Labrador Retriever** | 13.27 | 181,229 | 0.991 (1.020, 0.963) | 0.55 | 2.141 (1.385, 1.922) | <10^-6^ | 1.555 (1.705, 1.417) | <10^-6^ | 1.793 (1.967, 1.636) | <10^-6^ | 1.098 (1.144, 1.054) | 0.000007 | 1.279 (1.331, 1.229) | <10^-6^ | 1.255 (1.304, 1.208) | <10^-6^ |
| **Golden Retriever** | 21.73 | 99,111 | 1.031 (1.065, 0.998) | 0.063 | 3.172 (3.553, 2.832) | <10^-6^ | 1.726 (1.893, 1.574) | <10^-6^ | 2.025 (2.219, 1.848) | <10^-6^ | 0.989 (1.034, 0.945) | 0.64 | 1.022 (1.068, 0.978) | 0.33 | 1.161 (1.210, 1.113) | <10^-6^ |
| **German Shepherd** | 21.48 | 81,443 | 1.267 (1.312, 1.224) | <10^-6^ | 2.33 (2.605, 2.083) | <10^-6^ | 1.481 (1.631, 1.346) | <10^-6^ | 1.668 (1.836, 1.515) | <10^-6^ | 1.017 (1.069, 0.967) | 0.51 | 1.26 (1.321, 1.201) | <10^-6^ | 1.166 (1.222, 1.113) | <10^-6^ |
| **Rottweiler** | 22.65 | 75,450 | 1.154 (1.195, 1.114) | <10^-6^ | 1.986 (2.235, 1.764) | <10^-6^ | 1.395 (1.556, 1.251) | <10^-6^ | 1.489 (1.662, 1.333) | <10^-6^ | 0.972 (1.023, 0.934) | 0.28 | 1.072 (1.127, 1.020) | 0.0065 | 1.266 (1.329, 1.207) | <10^-6^ |
| **Australian Shepherd** | 6.06 | 27,163 | 1.058 (1.174, 0.953) | 0.29 | 2.719 (4.272, 1.730) | 0.00001 | 1.984 (3.000, 1.314) | 0.0011 | 1.917 (2.905, 1.266) | 0.002 | 0.92 (1.070, 0.791) | 0.28 | 1.081 (1.247, 0.937) | 0.28 | 1.117 (1.282, 0974) | 0.11 |
| **Poodle** | 12.55 | 18,519 | 0.981 (1.075, 0.894) | 0.68 | 1.482 (1.930, 1.139) | 0.0035 | 1.21 (1.489, 0.983) | 0.073 | 1.288 (1.586, 1.046) | 0.017 | 0.886 (1.004, 0.781) | 0.058 | 0.912 (1.035, 0.804) | 0.15 | 1.042 (1.169, 0.928) | 0.49 |
| **Bernese Mountain Dog** | 17.13 | 15,590 | 1.048 (1.142, 0.962) | 0.29 | 2.442 (3.629, 1.643) | 0.00001 | 1.661 (2.050, 1.345) | 0.000002 | 1.661 (2.043, 1.351) | 0.000002 | 1.049 (1.188, 0.927) | 0.45 | 1.167 (1.318, 1.034) | 0.013 | 1.164 (1.309, 1.035) | 0.011 |
| **Shetland Sheepdog** | 4.80 | 15,518 | 1.072 (1.255, 0.916) | 0.38 | 1.809 (3.022, 1.082) | 0.024 | 1.796 (2.763, 1.168) | 0.0078 | 1.904 (2.919, 1.242) | 0.0031 | 1.318 (1.639, 1.059) | 0.013 | 1.376 (1.690, 1.120) | 0.0024 | 1.086 (1.334, 0.884) | 0.43 |
| **Brittany** | 14.85 | 13,399 | 1.162 (1.283, 1.052) | 0.003 | 2.426 (3.791, 1.552) | 0.0001 | 1.538 (2.247, 1.052) | 0.026 | 1.56 (12.275, 1.070) | 0.021 | 1.162 (1.342, 1.007) | 0.04 | 1.115 (1.287, 0.967) | 0.14 | 1.279 (1.444, 1.113) | 0.00007 |
| **Newfoundland** | 27.64 | 12,085 | 1.065 (1.158, 0.979) | 0.14 | 5.844 (8.123, 4.204) | <10^-6^ | 1.982 (2.450, 1.603) | <10^-6^ | 2.078 (2.553, 1.691) | <10^-6^ | 0.857 (0.961, 0.765) | 0.008 | 1.008 (1.130, 0.900) | 0.90 | 1.267 (1.421, 1.128) | 0.000063 |
| **Akita** | 13.97 | 11,869 | 1.015 (1.132, 0.910) | 0.79 | 1.68 (2.245, 1.257) | 0.00046 | 0.881 (1.111, 0.698) | 0.29 | 1.03 (1.305, 0.813) | 0.81 | 0.997 (1.150, 0.865) | 0.97 | 1.136 (1.323, 0.975) | 0.10 | 0.922 (1.034, 0.777) | 0.35 |
| **Cocker Spaniel** | 9.60 | 11,697 | 0.834 (0.45, 0.735) | 0.0044 | 1.675 (2.568, 1.093) | 0.018 | 1.292 (1.874, 0.891) | 0.18 | 1.333 (1.933, 0.920) | 0.13 | 1.01 (1.213, 0.840) | 0.92 | 1.071 (1.280, 0.896) | 0.45 | 1.146 (1.360, 0.966) | 0.12 |
| **Doberman Pinscher** | 6.72 | 11,372 | 0.937 (1.090, 0.806) | 0.40 | 1.898 (3.060, 1.178) | 0.0085 | 1.603 (2.448, 1.049) | 0.029 | 1.463 (2.238, 0.956) | 0.079 | 1.19 (1.481, 0.956) | 0.12 | 1.129 (1.391, 0.917) | 0.25 | 0.993 (1.220, 0.809) | 0.95 |
| **English Springer Spaniel** | 13.43 | 11,159 | 0.932 (1.043, 0.832) | 0.22 | 2.531 (3.734, 1.716) | 0.000003 | 1.522 (2.018, 1.148) | 0.0035 | 1.587 (2.090, 1.206) | 0.0001 | 1.133 (1333, 0.965) | 0.13 | 1.13 (1.326, 0.964) | 0.13 | 1.22 (1.413, 1.054) | 0.008 |
| **German Shorthaired Pointer** | 4.47 | 10,839 | 0.926 (1.117, 0.768) | 0.42 | 3.953 (29.71, 0.526) | 0.18 | 1.64 (12.00, 0.224) | 0.63 | 2.151 (15.69, 0.295) | 0.45 | 1.415 (1.843, 1.086) | 0.010 | 1.416 (1.855, 1.081) | 0.011 | 1.102 (1.414, 0.859) | 0.45 |
| **Samoyed** | 11.22 | 10,500 | 0.981 (1.111, 0.866) | 0.76 | 1.564 (2.302, 1.063) | 0.023 | 0.928 (1.209, 0.713) | 0.58 | 0.988 (1.281, 0.762) | 0.93 | 0.96 (1.140, 0.808) | 0.64 | 0.96 (1.139, 0.809) | 0.64 | 1.032 (1.224, 0.871) | 0.71 |
| **Viszla** | 7.37 | 10,422 | 1.064 (1.241, 0.912) | 0.43 | 1.059 (2.466, 0.455) | 0.89 | 1.329 (2.871, 0.615) | 0.47 | 1.616 (3.481, 0.750) | 0.22 | 1.464 (1.811, 1.184) | 0.0004 | 0.272 (1.588, 1.027) | 0.028 | 1.12 (1.371, 0.915) | 0.27 |
| **Siberian Husky** | 2.04 | 9,854 | 1.316 (1.791, 0.966) | 0.081 | 4.739 (18.00, 1.247) | 0.022 | 3.665 (11.64, 1.154) | 0.028 | 4.08 (12.90, 1.290) | 0.017 | 1.632 (2.516, 1.067) | 0.024 | 1.495 (1.326, 0.961) | 0.074 | 1.436 (2.216, 0.930) | 0.10 |
| **Great Dane** | 13.57 | 9,647 | 0.999 (1.128, 0.885) | 0.99 | 4.272 (6.505, 2.806) | <10^-6^ | 2.146 (3.137, 1.468) | <10^-6^ | 2.681 (3.919, 1.833) | 0.00008 | 1.024 (1.218, 0.861) | 0.79 | 1.128 (1.336, 0.953) | 0.16 | 1.095 (1.286, 0.933) | 0.27 |
| **Border Collie** | 11.24 | 9,471 | 0.829 (0.943, 0.730) | 0.0043 | 1.736 (2.781, 1.084) | 0.022 | 1.049 (1.567, 0.703) | 0.82 | 1.235 (1.847, 0.826) | 0.30 | 1.042 (1.265, 0.860) | 0.68 | 1.007 (1.210, 0.838) | 0.94 | 1.127 (1.345, 0.945) | 0.18 |
| **Chespeake Bay Retriever** | 22.08 | 9,376 | 1.076 (1.192, 0.971) | 0.16 | 1.601 (2.361, 1.086) | 0.018 | 1.324 (1.770, 0.990) | 0.058 | 1.29 (1.721, 0.967) | 0.083 | 1.179 (1.367, 1.0-18) | 0.028 | 1.364 (1.572, 1.184) | 0.000017 | 1.463 (1.676, 1.277) | <10^-6^ |
| **Pembroke Welsh Corgi** | 21.97 | 9,345 | 1.133 (1.260, 1.018) | 0.022 | 1.392 (2.022, 0.958) | 0.083 | 1.258 (1.720, 0.920) | 0.15 | 1.127 (1.543, 0.823) | 0.46 | 0.774 (0.895, 0.670) | 0.0005 | 0.967 (1.114, 0.840) | 0.65 | 1.206 (1.379, 1.055) | 0.0061 |
| **Mastiff** | 22.03 | 9,319 | 1.107 (1.226, 0.999) | 0.052 | 2.538 (3.904, 1.650) | 0.0002 | 1.9 (2.824, 1.279) | 0.0015 | 1.876 (2.707, 1.260) | 0.0019 | 0.999 (1.150, 0.867) | 0.098 | 1.061 (1.219, 0.924) | 0.40 | 1.135 (1.305, 0.987) | 0.076 |
| **Rhodesian Ridgeback** | 4.41 | 8,865 | 1.128 (1.398, 0.911) | 0.27 | 3.495 (8.263, 1.478) | 0.0044 | 2.067 (4.697, 0.909) | 0.083 | 1.632 (3.743, 0.711) | 0.25 | 1.0902 (1.464, 0.814) | 0.56 | 1.134 (1.515, 0.849) | 0.39 | 0.974 (1.287, 0.737) | 0.85 |
| **Weimaraner** | 9.34 | 8,583 | 1.156 (1.351, 0.989) | 0.068 | 3.326 (6.472, 1.710) | 0.0004 | 2.512 (4.637, 1.361) | 0.0032 | 2.693 (4.967, 1.460) | 0.0015 | 1.268 (1.586, 1.014) | 0.038 | 1.622 (2.015, 1.306) | 0.000012 | 1.446 (1.776, 1.178) | 0.0043 |
| **Alaskan Malamute** | 11.76 | 8,514 | 1.32 (1.517, 1.148) | 0.00009 | 3.263 (6.580, 1.618) | 0.0009 | 1.403 (1.855, 1.061) | 0.018 | 1.307 (1.726, 0.890) | 0.059 | 1.104 (1.335,0.913) | 0.31 | 1.271 (1.537, 10.51) | 0.013 | 1.082 (1.307, 0.896) | 0.41 |
| **Chinese Shar-Pei** | 14.69 | 8,205 | 1.265 (1,441 1.110) | 0.0004 | 1.308 (2.478, 0.690) | 0.41 | 0.875 (1.597, 0.480) | 0.66 | 0.845 (1.546, 0.462) | 0.58 | 1.149 (1.355, 0.975) | 0.098 | 1.033 (1.244, 0.859) | 0.73 | 1.105 (1.331, 0.918) | 0.29 |
| **English Setter** | 15.17 | 7,728 | 0.986 (1.119, 0.868) | 0.83 | 0.987 (1.651, 0.591) | 0.96 | 0.984 (1.384, 0.700) | 0.93 | 1.152 (1.607, 0.826) | 0.41 | 0.911 (1.112, 0.747) | 0.36 | 1.303 (1.552, 1.094) | 0.003 | 1.252 (1.469, 1.068) | 0.006 |
| **Portuguese Water Dog** | 13.76 | 7,094 | 0.862 (0.989, 0.751) | 0.035 | 1.116 (1.826, 0.682) | 0.66 | 0.842 (1.208, 0.588) | 0.35 | 0.894 (1.273, 0.628) | 0.54 | 0.554 (0.690, 0.445) | <10^-6^ | 0.8644 (1.039, 0.719) | 0.12 | 0.85 (1.105, 0.712) | 0.072 |
| **Bouvier Des Flandres** | 16.06 | 6,483 | 0.913 (1.045, 0.797) | 0.19 | 1.239 (1.895, 0.810) | 0.32 | 0.998 (1.266, 0.787) | 0.99 | 0.924 (1.178, 0.725) | 0.53 | 0.892 (1.084, 0.735) | 0.25 | 0.826 (1004, 0.679) | 0.055 | 1.001 (1.194, 0.839) | 0.99 |
| **Irish Setter** | 11.02 | 6,401 | 0.946 (1.110, 0.806) | 0.49 | 1.644 (2.839, 0.952) | 0.074 | 1.091 (1.723, 0.691) | 0.71 | 1.027 (1.900, 0.767) | 0.42 | 0.959 (1.227, 0.750) | 0.74 | 1.15 (1.443, 0.917) | 0.23 | 1.183 (1.462, 0.956) | 0.12 |
| **Cavalier King Charles Spaniel** | 14.79 | 5,751 | 0.859 (0.999, 0.738) | 0.049 | 1.779 (3.458, 0.915) | 0.089 | 1.627 (2.759, 0.960) | 0.071 | 1.259 (2.142, 0.740) | 0.40 | 1.041 (1.284, 0.844) | 0.71 | 1.144 (1.412, 0.927) | 0.21 | 0.179 (1.451, 0.958) | 0.12 |
| **Old English Sheepdog** | 17.07 | 5,269 | 2.054 (3.534, 1.194) | 0.0093 | 1.683 (3.081, 0.919) | 0.092 | 0.981 (1.432, 0.671) | 0.92 | 1.405 (2.029, 0.972) | 0.07 | 0.859 (1.061,0.696) | 0.16 | 0.895 (1.092, 0.733) | 0.27 | 0.781 (0.951, 0.642) | 0.014 |
| **English Cocker Spaniel** | 6.22 | 5,132 | 1.24 (1.582, 0.971) | 0.085 | 3.985 (9.123, 1.741) | 0.0011 | 1.826 (3.646, 0.914) | 0.088 | 2.094 (4.141, 1.059) | 0.034 | 1.141 (1.597, 0.815) | 0.44 | 1.145 (1.611, 0.815) | 0.43 | 1.523 (2.072, 1.120) | 0.0073 |
| **Soft Coated Wheaten Terriier** | 5.49 | 4,799 | 1.159 (1.542, 0.872) | 0.31 | 1.511 (4.083, 0.559) | 0.42 | 1.242 (2.253, 0.685) | 0.48 | 1.607 (2.864, 0.902) | 0.11 | 1.2 (1.719, 0.838) | 0.32 | 1.085 (1.586, 0.743) | 0.67 | 1.166 (1.660, 0.820) | 0.39 |
| **Bull Mastiff** | 26.97 | 4,669 | 0.988 (1.127, 0.865) | 0.86 | 2.77 (3.963, 1.936) | <10^-6^ | 1.276 (1.697, 0.959) | 0.094 | 1.431 (1.897, 1.079) | 0.013 | 1.069 (1.288, 0.887) | 0.49 | 1.093 (1.315, 0.908) | 0.35 | 1.301 (1.560, 1.084) | 0.005 |
| **Great Pyrenees** | 9.78 | 4,506 | 1.196 (1.470, 0.973) | 0.089 | 2.968 (6.508, 1.354) | 0.0066 | 1.247 (2.190, 0.709) | 0.44 | 1.392 (2.447, 0.792) | 0.25 | 1.058 (1.431, 0.782) | 0.72 | 0.98 (1.318, 0.729) | 0.90 | 1.088 (1.451, 0.817) | 0.56 |
| **Airedale Terrier** | 12.71 | 4,489 | 0.991 (1.196, 0.820) | 0.92 | 1.662 (3.119, 0.886) | 0.11 | 1.464 (2.253, 0.952) | 0.083 | 1.65 (2.507, 1.086) | 0.019 | 0.206 (1.558, 0.934) | 0.15 | 1.175 (1.522, 0.908) | 0.22 | 1.222 (1.158, 0.965) | 0.096 |
| **Boxer** | 12.68 | 4,484 | 0.97 (1.163, 0.809) | 0.74 | 0.993 (1.617, 0.610) | 0.98 | 1.427 (2.028, 1.005) | 0.047 | 1.428 (2.020, 1.010) | 0.044 | 0.996 (1.273, 0.780) | 0.96 | 1.042 (1.333, 0.814) | 0.75 | 0.919 (1.170, 0.722) | 0.49 |
| **Flat Coated Retriever** | 4.75 | 4,388 | 1.533 (2.051, 1.146) | 0.004 | 1.839 (3.783, 0.894) | 0.098 | 0.785 (1.376, 0.448) | 0.40 | 0.929 (1.593, 0.542) | 0.79 | 1.308 (2.010, 0.851) | 0.22 | 1.34 (2.056, 0.874) | 0.18 | 1.274 (1.888, 0.680) | 0.23 |
| **Belgian Turveren** | 4.01 | 4,331 | 0.944 (1.280, 0.6966) | 0.71 | 2.242 (6.314, 0.796) | 0.13 | 1.525 (3.538, 0.657) | 0.33 | 1.213 (2.825, 0.521) | 0.65 | 1.004 (1.549, 0.650) | 0.99 | 0.801 (1.250, 0.514) | 0.33 | 0.847 (1.277, 0.562) | 0.43 |
| **Gordon Setter** | 20.08 | 4,251 | 0.776 (0.904, 0.667) | 0.0011 | 0.954 (1.669, 0.545) | 0.87 | 0.774 (1.134, 0.528) | 0.18 | 0.664 (.965, 0.457) | 0.032 | 1.11 (1.391, 0.885) | 0.37 | 1.132 (1.404, 0.913) | 0.26 | 1.199 (1.462, 0.983) | 0.073 |
| **Chow Chow** | 21.13 | 3,996 | 0.914 (1.067, 0.783) | 0.25 | 1.049 (1.784, 0.616) | 0.86 | 0.741 (1.094, 0.502) | 0.13 | 0.772 (1.146, 0.521) | 0.20 | 0.734 (0.898, 0.600) | 0.0027 | 0.938 (1.164, 0.757) | 0.56 | 0.86 (1.091, 0.679) | 0.21 |
| **German Wirehaired Pointer** | 8.27 | 3,681 | 0.858 (1.093, 0.674) | 0.21 | 4.671 (14.42, 1.513) | 0.0074 | 4.034 (8.870, 1.834) | 0.0005 | 4.442 (9.517, 2.073) | 0.0001 | 1.349 (1.950, 0.933) | 0.11 | 1.405 (1.980, 0.996) | 0.053 | 1.071 (1.479, 0.776) | 0.68 |
| **Keeshond** | 6.90 | 3,500 | 1.234 (1.625, 0.937) | 0.13 | 0.698 (2.437, 0.200) | 0.57 | 1.825 (3.973, 0.838) | 0.13 | 1.738 (3.809, 0.793) | 0.17 | 0.855 (1.230, 0.595) | 0.40 | 0.802 (1.174, 0.548) | 0.26 | 0.953 (1.365, 0.665) | 0.79 |
| **Bearded Collie** | 6.12 | 3,384 | 0.612 (0.813, 0.461) | 0.0007 | 24.99 (190.5, 3.279) | 0.0019 | 7.882 (57.19, 1.086) | 0.041 | 8.085 (58.50, 1.117) | 0.038 | 0.604 (0.934, 0.321) | 0.023 | 0.417 (0.652, 0.267) | 0.0001 | 0.892 (1.261, 0.630) | 0.52 |
| **Australian Cattle Dog** | 17.03 | 3,182 | 0.954 (1.153, 0.789) | 0.63 | 1.772 (3.261, 0.963) | 0.066 | 1.498 (2.566, 0.875) | 0.14 | 1.342 (2.327, 0.774) | 0.29 | 0.794 (1.057, 0.597) | 0.11 | 1.23 (1.595, 0.948) | 0.12 | 1.188 (1.529, 0.922) | 0.18 |
| **Standard Schnauzer** | 8.40 | 3,162 | 1.1 (1.431, 0.846) | 0.48 | 2.154 (5.546, 0.837) | 0.11 | 1.094 (2.301, 0.520) | 0.81 | 1.235 (2.597, 0.587) | 0.58 | 0.776 (1.114, 0.541) | 0.17 | 0.852 (1.221, 0.594) | 0.38 | 0.879 (1.223, 0.631) | 0.44 |
| **Havanese** | 11.67 | 3,067 | 1.191 (1.513, 0.938) | 0.15 | 0.712 (1.425, 0.356) | 0.34 | 0.715 (1.347, 0.376) | 0.29 | 0.736 (1.398, 0.387) | 0.35 | 0.7677 (1.080, 0.559) | 0.13 | 1.141 (1.543, 0.844) | 0.39 | 0.947 (1.295, 0.693) | 0.74 |
| **American Pit Bull Terrier** | 29.68 | 2,953 | 0.89 (1.051, 0.754) | 0.17 | 1.19 (1.881, 0.666) | 0.67 | 0.587 (0.9409, 0.366) | 0.023 | 0.694 (1.120, 0.430) | 0.13 | 1.107 (1.393, 0.880) | 0.38 | 1.241 (1.547, 0.995) | 0.056 | 1.094 (1.359, 0.880) | 0.42 |
| **Giant Schnauzer** | 18.41 | 2,946 | 1.503 (1.835, 1.231) | 0.00006 | 2.471 (4.908, 1.244) | 0.0097 | 2.185 (4.053, 1.178) | 0.013 | 1.61 (2.983, 0.869) | 0.13 | 1.149 (1.511, 0.865) | 0.32 | 1.22 (1.593, 0.934) | 0.14 | 1.084 (1.405, 0.838) | 0.54 |
| **Tibetan Terrier** | 6.74 | 2,921 | 0.85 (1.145, 0.632) | 0.29 | 2.591 (8.064, 0.833) | 0.10 | 1.476 (4.125, 0.528) | 0.46 | 1.878 (5.216, 0.676) | 0.23 | 0.912 (1.412, 0.590) | 0.68 | 1.21 (1.829, 0.801) | 0.37 | 1.36 (2.017, 0.917) | 0.13 |
| **Dalmatian** | 4.08 | 2,882 | 1.257 (1.860, 0.850) | 0.25 | 0.78 (1.994, 0.305) | 0.60 | 0.52 (1.042, 0.259) | 0.065 | 0.351 (0.732, 0.168) | 0.0053 | 1.595 (2.172, 0.856) | 0.14 | 1.558 (2.917, 0.832) | 0.17 | 2.34 (4.106, 1.334) | 0.003 |
| **Bichon Frise** | 8.01 | 2,834 | 1.876 (2.579, 1.364) | 0.00011 | 2.065 (4.717, 0.904) | 0.085 | 1.144 (2.179, 0.601) | 0.68 | 1.033 (1.987, 0.537) | 0.92 | 0.962 (1.426, 0.648) | 0.85 | 1.001 (1.491, 0.672) | 0.99 | 1.237 (1.791, 0.854) | 0.26 |
| **Shiba Inu** | 6.10 | 2,829 | 0.985 (1.38, 0.715) | 0.93 | 1.821 (4.252, 0.780) | 0.17 | 0.748 (1.380, 0.406) | 0.35 | 1.364 (2.428, 0.766) | 0.29 | 0.88 (1.318, 0.588) | 0.54 | 0.952 (1.475, 0.715) | 0.83 | 0.903 (1.419, 0.575) | 0.66 |
| **Belgian Sheepdog** | 3.38 | 2,792 | 0.794 (1.197, 0.526) | 0.27 | 3.175 (10.43, 0.966) | 0.057 | 1.016 (2.321, ).445) | 0.97 | 1.418 (3.171, 0.634) | 0.39 | 1.175 (2.207, 0.626) | 0.62 | 1.16 (2.165, 0.622) | 0.64 | 1.569 (2.755, 0.894) | 0.12 |
| **Afghan Hound** | 7.06 | 2,781 | 0.613 (0.821, 0.457) | 0.0011 | 7.348 (56.68, 0.953) | 0.056 | 5.316 (38.77, 0.729) | 0.099 | 7.746 (56.32, 10.66) | 0.043 | 1.383 (2.097, 0.912) | 0.13 | 1.088 (1.689, 0.700) | 0.71 | 1.058 (1.577, 0.710) | 0.78 |
| **Boykin Spaniel** | 38.19 | 2,655 | 0.789 (0.925, 0.673) | 0.0034 | 3.824 (33.01, 0.443) | 0.22 | 3.783 (31.55, 0.454) | 0.22 | 3.552 (29.96, 0.421) | 0.24 | 0.998 (1.253, 0.795) | 0.99 | 1.151 (1.447, 0.916) | 0.23 | 1.073 (1.330, 0.865) | 0.52 |
| **Greater Swiss Mountain Dog** | 20.67 | 2,487 | 0.77 (0.938, 0.632) | 0.0095 | 6.94 (30.45, 1.582) | 0.01 | 5.042 (20.94, 1.214) | 0.026 | 7.043 (29.24, 1.696) | 0.007 | 1.12 (1.493, 0.841) | 0.44 | 0.941 (1.233, 0.719) | 0.66 | 1.051 (1.379, 0.802) | 0.72 |
| **Belgian Malinois** | 5.65 | 2,478 | 0.993 (1.400, 0.704) | 0.97 | 2.048 (7.048, 0.595) | 0.26 | 0.819 (2.703, 0.248) | 0.74 | 1.263 (4.185, 0.381) | 0.71 | 1.431 (2.332, 0.878) | 0.15 | 1.027 (1.704, 0.619) | 0.92 | 0.907 (1.427, 0.558) | 0.69 |
| **Norwegian Elkhound** | 19.96 | 2,437 | 0.96 (1.176, 0.784) | 0.69 | 0.764 (3.147, 0.185) | 0.71 | 0.854 (1.696, 0.430) | 0.65 | 0.906 (1.795, 0.437) | 0.78 | 0.98 (1.295, 0.742) | 0.89 | 1.044 (1.371, 0.794) | 0.76 | 0.963 (1.277, 0.727) | 0.79 |
| **Border Terrier** | 4.23 | 2,364 | 1.851 (2.999, 1.143) | 0.012 | 4.766 (39.89, 0.570) | 0.15 | 4.128 (15.79, 0.287) | 0.46 | 3.211 (23.67, 0.436) | 0.25 | 1.332 (2.578, 0.741) | 0.31 | 1.883 (3.424, 1.036) | 0.038 | 1.482 (2.714, 0.810) | 0.20 |
| **Basenji** | 3.58 | 2,119 | 1.271 (2.045, 0.790) | 0.32 | 4.242 (36.28, 0.496) | 0.19 | 2.044 (15.21, 0.275) | 0.49 | 2.604 (19.43, 0.349) | 0.35 | 5.52 (41.40, 0.637) | 0.096 | 4.682 (34.2, 0.641) | 0.13 | 2.132 (34.7, 0.131) | 0.60 |
| **Bloodhound** | 29.32 | 2,005 | 1.611 (1.974, 1.316) | 0.000004 | 1.043 (2.054, 0.530) | 0.90 | 0.854 (1.552, 0.470) | 0.61 | 0.885 (1.616, 0.485) | 0.69 | 0.961 (1.272, 0.726) | 0.78 | 1.127 (1.471, 0.864) | 0.38 | 0.8 (1.045, 0.612) | 0.10 |
| **Collie** | 3.35 | 1,968 | 0.747 (1.226, 0.455) | 0.25 | - | - | - | - | - | - | 0.455 (0.999, 0.207 | 0.049 | 0.687 (1.326, 0.356) | 0.26 | 0.708 (1.334, 0.376) | 0.29 |
| **Briard** | 15.73 | 1,881 | 0.871 (1.119, 0.677) | 0.28 | 4.345 (17.00, 1.112) | 0.035 | 2.278 (7.517, 0.690) | 0.18 | 2.276 (7.474, 0.693) | 0.18 | 1.048 (1.946, 0.734) | 0.80 | 0.941 (1.340, 0.660) | 0.80 | 0.889 (1.262, 0.6260 | 0.51 |
| **Welsh Springer Spaniel** | 11.47 | 1,807 | 0.985 (1.322, 0.734) | 0.92 | 4.399 (37.73, 0.513) | 0.18 | 3.436 (25.58, 0.462) | 0.23 | 3.686 (27.45, 0.495) | 0.20 | 1.07 (1.637, 0.700) | 0.75 | 1.042 (1.607, 0.676) | 0.85 | 1.107 (1.652, 0.742) | 0.62 |
| **Nova Scotia Duck Tolling Retriever** | 7.17 | 1,755 | 1.265 (1.850, 0.865) | 0.23 | 2.245 (5.015, 1.006) | 0.048 | 1.351 (2.296, 0.797) | 0.26 | 1.188 (1.910, 0.739) | 0.48 | 1.039 (1.835, 0.588) | 0.89 | 1.154 (1.981, 0.673) | 0.60 | 1.281 (2.146, 0.765) | 0.35 |
| **Leonberger** | 15.90 | 1,711 | 0.857 (1.115, 0.659) | 0.25 | 0.402 (3.225, 0.51) | 0.39 | 1.258 (2.256, 0.701) | 0.44 | 1.642 (2.840, 0.950) | 0.44 | 0.795 (1.132, 0.558) | 0.20 | 0.724 (1.035, 0.506) | 0.076 | 0.54 (0.781, 0.374) | 0.001 |
| **Miniature Australian Shepherd** | 8.19 | 1,649 | 1.34 (2069, 0.947) | 0.092 | 0.466 (2.262, 0.096) | 0.34 | 0.579 (1.716, 0.195) | 0.32 | 0.52 (1.548, 0.174) | 0.24 | 1.255 (2.315, 0.736) | 0.40 | 1.637 (2.692, 0.996) | 0.052 | 1.229 (2.065, 0.727) | 0.44 |
| **Anatolian Shepherd** | 11.40 | 1,641 | 1.115 (1.529, 0.813) | 0.50 | 1.385 (6.650, 0.288) | 0.68 | 1.201 (5.253, 0.275) | 0.81 | 1.015 (4.533, 0.227) | 0.98 | 0.945 (1.565, 0.561) | 0.83 | 1.375 (2.157, 0.877) | 0.17 | 1.372 (2.155, 0.875) | 0.17 |
| **Wirehaired Pointing Griffon** | 9.13 | 1,620 | 0.979 (1.389, 0.690) | 0.91 | 1.61 (19.19, 0.136) | 0.71 | 2.479 (10.68, 0.576) | 0.22 | 2.301 (9.621, 0.550) | 0.25 | 0.975 (1.822, 0.522) | 0.94 | 1.597 (2.601, 0.980) | 0.060 | 1.065 (1.706, 0.665) | 0.79 |
| **Cardigan Welsh Corgi** | 25.05 | 1,589 | 1.042 (1.318, 0.823) | 0.73 | 4.112 (14.79, 1.143) | 0.03 | 3.08 (10.28, 0.923) | 0.067 | 3.431 (11.46, 1.028) | 0.045 | 0.623 (0.868, 0.448) | 0.005 | 0.913 (1.266, 0.659) | 0.59 | 1.223 (1.659, 0.901) | 0.20 |
| **Hybrid** | 20.43 | 1,536 | 0.87 (1.151, 0.658) | 0.33 | 1.215 (3.125, 0.478) | 0.69 | 1.46 (3.104, 0.721) | 0.28 | 1.531 (3.186, 0.736) | 0.25 | 0.946 (1.355, 0.660) | 0.76 | 0.904 (1.290, 0.634) | 0.58 | 0.936 (1.315, 0.666) | 0.70 |
| **American Bulldog** | 39.83 | 1,445 | 0.915 (1.131, 0.741) | 0.41 | 0.815 (1.898, 0.350) | 0.63 | 0.884 (1.932, 0.404) | 0.76 | 1.137 (2.501, 0.527) | 0.75 | 0.938 (1.260, 0.698) | 0.67 | 1.12 (1.516, 0.828) | 0.46 | (1.035 (1.389, 0.771) | 0.82 |
| **Kuvasz** | 18.49 | 1,370 | 1.233 (1.643, 0.925) | 0.15 | 3.641 (10.54, 1.258) | 0.017 | 1.36 (2.732, 0.678) | 0.39 | 1.483 (2.988, 0.736) | 0.27 | 0.596 (0.933, 0.381) | 0.024 | 1.002 (1.472, 0682) | 0.99 | 1.314 (1.914, 0.902) | 0.15 |
| **Irish Wolfhound** | 4.98 | 1,282 | 1.416 (2.504, 0.800) | 0.23 | 2.976 (12.90, 0.686) | 0.15 | 0.733 (2.025, 0.244) | 0.58 | 0.964 (2.811, 0.331) | 0.95 | 1.312 (2.942, 0.585) | 0.51 | 1.644 (3.438, 0.785) | 0.18 | 1.402 (2.947, 0.667) | 0.37 |
| **Pointer** | 8.74 | 1,234 | 0.875 (1.303, 0.588) | 0.51 | 2.861 (26.88, 0.305) | 0.36 | 3.156 (23.54, 0.423) | 0.26 | 3.08 (23.12, 0.410) | 0.27 | 0.947 (1.651, 0.543) | 0.85 | 0.753 (1.273, 0.445) | 0.29 | 0.569 (0.984, 0.329) | 0.044 |
| **Saint Bernard** | 52.14 | 1,222 | 1.445 (1.826, 1.143) | 0.002 | 3.017 (10.936, 0.833) | 0.093 | 0.729 (2.824, 1.058) | 0.029 | 1.705 (2.738, 1.062) | 0.027 | 0.924 (1.287, 0.664) | 0.64 | 1.135 (1.597, 0.806) | 0.47 | 1.21 (1.675, 0.873) | 0.25 |
| **French Bulldog** | 34.11 | 1,111 | 1.4 (1.810, 1.084) | 0.010 | 1.101 (3.145, 0.385) | 0.86 | 1.811 (4.335, 0.756) | 0.18 | 1.723 (4.138, 0.718) | 0.22 | 0.947 (1.354, 0.662) | 0.77 | 1.229 (1.785, 0.846) | 0.28 | 1.29 (1.831, 0.909) | 0.15 |
| **Spinone Italiano** | 18.42 | 1,073 | 1.437 (2.003, 1.030) | 0.033 | 0.933 (6.289, 0.138) | 0.94 | 1.907 (8.556, 0.425) | 0.40 | 1.663 (7.439, 0.372) | 0.51 | 1.293 (2.098, 0.797) | 0.30 | 1.594 (2.494,1.018) | 0.041 | 1.522 (2.360, 0.982) | 0.06 |
| **Puli** | 10.45 | 1,062 | 0.823 (1.224, 0.553) | 0.34 | 1.442 (5.238, 0.397) | 0.58 | 0.727 (1.805, 0.293) | 0.49 | 0.992 (2.443, 0.403) | 0.99 | 1.797 (3.354, 0.963) | 0.065 | 1.501 (2.822, 0.798) | 0.21 | 1.217 (2.319, 0.639) | 0.55 |
| **Irish Water Spaniel** | 12.26 | 1,051 | 1.285 (1.881, 0.877) | 0.20 | 0.565 (2.789, 0.114) | 0.48 | 0.453 (0.907, 0.226) | 0.025 | 0.618 (1.161, 0.329) | 0.13 | 1.279 (2.381, 0.687) | 0.44 | 1.447 (2.545, 0.823) | 0.20 | 1.595 (2.702, 0.941) | 0.083 |
|  |  |  |  |  |  |  |  |  |  |  |  |  |  |  |  |  |
| **Dogs with n > 100 and CHD prevalence > 156%** |  |  | **Sex** |  | **Latitude** |  |  |  |  |  | **Season of birth** |  |  |  |  |  |
|  |  |  | **Female** |  | **<30° N** |  | **30-39° N** |  | **40-49° N** |  | **Autumn** |  | **Winter** |  | **Spring** |  |
|  | **% CHD** | **n** | **OR (95%CI)** | **p value** | **OR (95%CI)** | **p value** | **OR (95%CI)** | **p value** | **OR (95%CI)** | **p value** | **OR (95%CI)** | **p value** | **OR (95%CI)** | **p value** | **OR (95%CI)** | **p value** |
| **Bulldog** | 77.67 | 514 | 0.836 (1.275, 0.548) | 0.41 | 2.111 (12.41, 0.359) | 0.41 | 0.85 (3.198, 0.226) | 0.81 | 1.248 (4.771, 0.327) | 0.75 | 1.196 (2.172, 0.658) | 0.56 | 1.14 (2.021, 0.6433) | 0.65 | 1.419 (2.540, 0.793) | 0.24 |
| **Pug** | 73.14 | 484 | 0.94 (1.422, 0.621) | 0.77 | 0.83 (3.253, 0.212) | 0.79 | 2.818 (4.826, 1.646) | 0.00016 | 2.198 (3.596, 1.343) | 0.0017 | 1.275 (2.344, 0.694) | 0.43 | 1.286 (2.348, 0.704) | 0.41 | 1.128 (1.988, 0.641) | 0.68 |
| **Dogue de Bordeaux** | 65.27 | 428 | 0.895 (1.370, 0.591) | 0.62 | 2.175 (8.954, 0.528) | 0.28 | 1.106 (3.340, 0.367) | 0.86 | 1.797 (5.460, 0.591) | 0.30 | 1.113 (1.934, 0.640) | 0.71 | 1.443 (2.556, 0.814) | 0.21 | 1.716 (3.123, 0.943) | 0.077 |
| **Otterhound** | 54.55 | 363 | 1.393 (2.117, 0.917) | 0.12 | 0.571 (2.167, 0.151) | 0.41 | 0.791 (2.009, 0.312) | 0.63 | 1.081 (2.661, 0.439) | 0.87 | 1.026 (1.913, 0.550) | 0.94 | 0.884 (1.667, 0.469) | 0.70 | 0.834 (1.418, 0.490) | 0.50 |
| **Bourboel** | 52.24 | 134 | 1.079 (2.175, 0.540) | 0.83 | 1.812 (18.23, 0.180) | 0.61 | 1.745 (11.62, 0.260) | 0.57 | 1.788 (12.47, 0.256) | 0.56 | 1.077 (3.036, 0.328) | 0.89 | 1.922 (5.362, 0.689) | 0.21 | 1.278 (3.461, 0.472) | 0.63 |
| **Neopolitan Mastiff** | 51.52 | 131 | 0.654 (1.529, 0.372) | 0.44 | 3.702 (58.77, 0.233) | 0.35 | 4.027 (38.60, 0.420) | 0.23 | 4.907 (47.94, 0.502) | 0.23 | 0.909 (2.423, 0.341) | 0.85 | 1.293 (3.565, 0.469) | 0.62 | 0.894 (2.315, 0.345) | 0.62 |
| **Bassett Hound** | 50.00 | 130 | 1.051 (2.256, 0.490) | 0.89 | 3.954 (42.96, 0.364) | 0.26 | 1.213 (2.523,0.583) | 0.61 | - - | - | 1.866 (5.283, 0.659) | 0.24 | 2.612 (7.151, 0.954) | 0.062 | 0.742 (2.116, 0.260) | 0.58 |
| **Black Russian Terrier** | 47.45 | 548 | 1.663 (2.355, 1.174) | 0.004 | 1.292 (9.439, 0.556) | 0.25 | 1.554 (5.418, 0.446) | 0.49 | 2.58 (8.991, 0.739) | 0.14 | 0.589 (0.984, 0.353) | 0.043 | 0.693 (1.127, 0.426) | 0.14 | 0.788 (1.266, 0.490) | 0.32 |
| **Sussex Spaniel** | 47.35 | 226 | 2.466 (4.283, 1.419) | 0.0014 | - | - | - | - | - | - | 0.924 (1.994, 0.748) | 0.84 | 0.85 (1.778, 0.406) | 0.67 | 0.937 (1.975, 0.445) | 0.86 |
| **Argentine Dogo** | 44.51 | 182 | 1.309 (2.427, 0.708) | 0.39 | 0.95 (3.911, 0.231) | 0.94 | 0.843 (1.567, 0.453) | 0.59 | - - | - | 1.966 (4.517, 0.856) | 0.11 | 1.843 (4.085, 0.832) | 0.13 | 0.034 (1.520, 0.187) | 0.24 |
| **Cane Corso** | 44.47 | 876 | 0.836 (1.101, 0.634) | 0.20 | 1.137 (2.356, 0.549) | 0.73 | 1.496 (2.717, 0.823) | 0.19 | 1.662 (3.016, 0.915) | 0.095 | 1.233 (1.807, 0.841) | 0.28 | 1.099 (1.593, 0.758) | 0.62 | 1.204 (1.775, 0.817) | 0.35 |
| **Perro de Presa Canario** | 41.67 | 168 | 0.733 (1.387, 0.387) | 0.34 | 5.517 (64.58, 0.471) | 0.17 | 3.64 (35.238, 0.376) | 0.26 | 2.732 (26.74, 0.279) | 0.39 | 1.205 (2.889, 0.503) | 0.67 | 0.806 (1.997, 0.325) | 0.64 | 1.071 (2.552, 0.450) | 0.88 |
| **Norfolk Terrier** | 40.83 | 240 | 2.259 (3.899, 1.309) | 0.003 | 5.542 (96.34, 0.319) | 0.24 | 1.393 (6.239, 0.311) | 0.66 | 1.089 (4.721,0.251) | 0.91 | 0.949 (2.146, 0.420) | 0.89 | 0.769 (1.593, 0.3161) | 0.48 | 1.429 (2.849, 0.716) | 0.31 |
| **Glen of Imaal Terrier** | 37.78 | 135 | 1.514 (3.265, 0.702) | 0.29 | 0.515 (1.851, 0.144) | 0.31 | 0.723 (1.723, 0.304) | 0.46 | - - | - | 1.027 (2.998, 0.362) | 0.96 | 0.591 (1.733, 0.202) | 0.34 | 1.005 (2.504, 0.403) | 0.99 |
| **Spanish Water Dog** | 35.96 | 114 | 0.477 (1.125, 0.203) | 0.091 | 1.975 (15.82, 0.247) | 0.52 | 3.181 (18.99, 0.533) | 0.20 | 1.744 (9.493, 0.320) | 0.52 | 1.362 (4.123, 0.450) | 0.58 | 0.685 (1.943, 0.241) | 0.48 | 0.396 (1.368, 0.115) | 0.14 |
| **Fila Brasiliero** | 32.35 | 473 | 1.149 (1.705, 0.774) | 0.49 | 1.029 (6.049, 0.175) | 0.97 | 0.422 (2.173, 0.082) | 0.31 | 0.34 (1.794, 0.065) | 0.20 | 1.225 (2.174, 0.690) | 0.49 | 1.114 (1.970, 0.630) | 0.71 | 0.247 (2.201, 0.706) | 0.45 |
| **Lagotto Ramagnolo** | 29.41 | 170 | 1.813 (3.811, 0.862) | 0.12 | 9.782 (65.17, 1.468) | 0.018 | 3.442 (11.265, 1.052) | 0.041 | 1.806 (6.416, 0.508) | 0.36 | 1.138 (3.222, 0.402) | 0.81 | 2.08 (6.290, 0.688) | 0.19 | 1.291 (3.427, 0.487) | 0.61 |
| **English Shepherd** | 25.38 | 331 | 0.918 (1.542, 0.546) | 0.75 | 0.702 (9.301, 0.053) | 0.79 | 0.617 (7.239, 0.053) | 0.70 | 0.535 (6.199, 0.046) | 0.62 | 0.575 (1.140, 0.290) | 0.11 | 0.583 (1.186, 0.286) | 0.14 | 0.54 (1.083, 0.270) | 0.083 |
| **Shih Tzu** | 23.96 | 384 | 1.815 (3.052, 1.080) | 0.024 | 0.467 (2.944, 0.074) | 0.42 | 0.449 (2.632, 0.077) | 0.42 | 0.688 (4.030, 0.117) | 0.38 | 0.603 (1.158, 0.314) | 0.13 | 0.737 (1.509, 0.360) | 0.40 | 0.71 (1.352, 0.373) | 0.30 |
| **Louisiana Catahoula Leopard** | 23.09 | 472 | 0.865 (1.351, 0.554) | 0.52 | 6.59 (53.88, 0.806) | 0.079 | 2.471 (20.05, 0.304) | 0.40 | 2.455 (20.81, 0.290) | 0.41 | 2.056 (4.151, 1.019) | 0.044 | 2.316 (4.498, 1.192) | 0.013 | 2.135 (4.042, 1.128) | 0.019 |
| **Havana Silk Dog** | 22.73 | 154 | 1.645 (4.235, 0.639) | 0.30 | - | - | - | - | - | - | 0.369 (1.368, 0.994) | 0.14 | 1.466 (4.316, 0.498) | 0.49 | 0.633 (1.834, 0.218) | 0.40 |
| **Berger de Picard** | 22.56 | 133 | 0.84 (2.056, 0.343) | 0.70 | - | - | - | - | - | - | 2.807 (9.648,0.817) | 0.10 | 0.781 (3.016, 0.203) | 0.72 | 3.471 (12.15, 0.992) | 0.0952 |
| **Chinook** | 20.53 | 570 | 0.658 (0.997, 0.434) | 0.048 | 0.961 (11.41, 0.081) | 0.68 | 0.728 (6.483, 0.082) | 0.049 | 1.566 (13.40, 0.183) | 0.18 | 0.685 (1.194, 0.393) | 0.18 | 0.992 (1.787, 0.550) | 0.98 | 0.511 (0.900, 0.290) | 0.02 |
| **Field Spaniel** | 20.22 | 989 | 1.505 (2.087, 1.085) | 0.014 | - | - | - | - | - | - | 0.997 (1.616, 0.619) | 0.99 | 1.14 (1.822, 0.713) | 0.58 | 1.782 (2.798, 1.135) | 0.012 |
| **Shiloh Shepherd** | 20.03 | 749 | 1.288 (1.867, 0.888) | 0.18 | 3.367 (16.20, 0.700) | 0.13 | 1.546 (5.451, 0.438) | 0.50 | 1.67 (5.776, 0.438) | 0.42 | 0.82 (1.370, 0.491) | 0.45 | 1.266 (2.0935, 0.788) | 0.33 | 0.523 (0.889, 0.308) | 0.017 |
| **Polish Tatra Sheepdog** | 20.00 | 140 | 3.36 (9.947, 11.35) | 0.029 | - | - | - | - | - | - | 1.141 (4.007, 0.325) | 0.84 | 0.737 (3.600, 0.151) | 0.71 | 0.992 (3.214, 0.306) | 0.99 |
| **Beagle** | 19.92 | 732 | 0.878 (1.270, 0.608) | 0.49 | 1.329 (3.330, 0.530) | 0.54 | 1.504 (3.032, 0.746) | 0.25 | 0.94 (1.934, 0.457) | 0.87 | 0.797 (1.364, 0.465) | 0.41 | 0.848 (1.426, 0.505) | 0.53 | 0.807 (1.285, 0.507) | 0.37 |
| **Pyrenean Shepherd** | 19.69 | 127 | 1.078 (2.803, 0.415) | 0.88 | - | - | - | - | - | - | 0.272 (0.914, 0.081) | 0.035 | 0.366 (1.247, 0.107) | 0.11 | 0.134 (0.675, 0.026) | 0.015 |
| **Affenpinscher** | 18.95 | 285 | 1.078 (2.015, 0.576) | 0.82 | 0.746 (4.394, 0.127) | 0.75 | 1.136 (5.699, 0.228) | 0.88 | 0.451 (2.501, 0.081) | 0.36 | 0.34 (0.826, 0.140) | 0.017 | 0.711 (1.592, 0.318) | 0.41 | 0.333 (0.765, 0.145) | 0.0096 |
| **Staffordshire Bull Terrier** | 18.83 | 513 | 1.045 (1.651, 0.665) | 0.84 | 0.719 (2.698, 0.192) | 0.63 | 0.847 (2.691, 0.267) | 0.78 | 1.052 (3.413, 0.324) | 0.93 | 0.816 (1.549, 0.430) | 0.53 | 0.897 (1.706, 0.471) | 0.74 | 1.057 (1.953, 0.572) | 0.86 |
| **Polish Lowland Sheepdog** | 18.41 | 427 | 0.708 (1.165, 0.430) | 0.17 | 1.236 (6.877, 0.222) | 0.81 | 1.274 (6.089, 0.266) | 0.76 | 1.592 (7.369, 0.344) | 0.55 | 0.955 (2.033, 0.449) | 0.91 | 1.251 (2.449, 0.639) | 0.51 | 1.183 (2.310, 0.606) | 0.62 |
| **Entlebucher** | 17.45 | 298 | 1.614 (3.109, 0.838) | 0.15 | 26.72 (476.3, 1.499) | 0.025 | 2.754 (1.274, 0.595) | 0.19 | 2.85 (12.90, 0.629) | 0.17 | 1.56 (4.068, 0.621) | 0.33 | 1.278 (3.715, 0.440) | 0.65 | 2.377 (5.522, 1.023) | 0.044 |
| **Curly Coated Retriever** | 17.43 | 918 | 1.372 (1.946, 0.967) | 0.076 | 0.615 (2.620, 0.144) | 0.51 | 0.544 (2.121, 0.140) | 0.38 | 0.591 (2.306, 0.151) | 0.45 | 0.996 (1.615, 0.614) | 0.99 | 1.144 (1.875, 0.698) | 0.59 | 0.853 (1.372, 0.536) | 0.52 |
| **Tibetan Mastiff** | 17.35 | 853 | 0.741 (1.062, 0.518) | 0.10 | 1.292 (8.355, 0.200) | 0.79 | 0.416 (0.924, 0.187) | 0.031 | 0.566 (1.249, 0.256) | 0.16 | - - | - | - - | - | - - | - |
| **Barbet** | 16.67 | 102 | 0.867 (2.603, 0.289) | 0.80 | - | - | - | - | - | - | 0.206 (1.962, 0.022) | 0.17 | 0.49 (2.169, 0.111) | 0.35 | 0.857 (3.200, 0.229) | 0.82 |
| **Labradoodle** | 16.35 | 318 | 1.234 (2.387, 0.638) | 0.53 | 0.391 (2.110, 0.073) | 0.27 | 0.797 (1.946, 0.326) | 0.62 | 0.398 (1.082, 0.146) | 0.071 | 1.125 (2.577, 0.491) | 0.78 | 0.941 (2.128, 0.416) | 0.88 | 0.747 (1.773, 0.314) | 0.51 |
| **Norwich Terrier** | 16.29 | 792 | 0.906 (1.335, 0.615) | 0.62 | 14.24 (88.7, 2.285) | 0.0044 | 3.491 (14.87, 0.820) | 0.091 | 4.847 (20.58, 1.141) | 0.032 | 0.804 (1.381, 0.438) | 0.43 | 0.9 (1.521, 0.532) | 0.69 | 0.702 (1.188, 0.415) | 0.19 |
| **Black and Tan Coonhound** | 16.01 | 456 | 1.627 (2.854, 0.927) | 0.089 | 0.556 (2.850, 0.109) | 0.48 | 0.586 (2.300, 0.150) | 0.44 | 0.419 (1.695, 0.103) | 0.22 | 0.993 (2.090, 0.472) | 0.98 | 0.855 (1.794, 0.408) | 0.68 | 1.22 (2.342, 0.636) | 0.55 |

The cells with statistically significant p values are shaded in yellow

The reference groups were male, latitude > 50° N, and summer
